# Why sensitive bacteria are resistant to hospital infection control

**DOI:** 10.12688/wellcomeopenres.11033.2

**Published:** 2017-11-22

**Authors:** Esther van Kleef, Nantasit Luangasanatip, Marc J Bonten, Ben S Cooper

**Affiliations:** 1 Julius Centre for Health Sciences and Primary Care, University Medical Center Utrecht, Huispost nr. STR 6.131, P.O. Box 85500, Utrecht, Netherlands; 2 Modelling and Economics Unit, National Infection Service, Public Health England, 61 Colindale Avenue, London, NW9 5EQ, UK; 3 Mahidol-Oxford Tropical Medicine Research Unit, Faculty of Tropical Medicine, Mahidol University, 420/6 Rajvithi Road, Tungphyathai, Bangkok, 10400, Thailand; 4 Department of Infectious Disease Epidemiology, London School of Hygiene and Tropical Medicine, London, WC1E 7HT, UK; 5 Department of Medical Microbiology, University Medical Centre Utrecht, P.O. 85500, Utrecht, Netherlands; 6 Nuffield Department of Medicine, University of Oxford, Old road, Oxford, OX3 7LF, UK

**Keywords:** mathematical modelling, nosocomial infections, hospital infection control, Clostridium difficile, Staphylococcus aureus, MRSA, hand hygiene, antibiotic resistance

## Abstract

**Background**: Large reductions in the incidence of antibiotic-resistant strains of *Staphylococcus aureus* and *Clostridium difficile* have been observed in response to multifaceted hospital-based interventions. Reductions in antibiotic-sensitive strains have been smaller or non-existent. It has been argued that since infection control measures, such as hand hygiene, should affect resistant and sensitive strains equally, observed changes must have largely resulted from other factors, including changes in antibiotic use. We used a mathematical model to test the validity of this reasoning.

**Methods**: We developed a mechanistic model of resistant and sensitive strains in a hospital and its catchment area. We assumed the resistant strain had a competitive advantage in the hospital and the sensitive strain an advantage in the community. We simulated a hospital hand hygiene intervention that directly affected resistant and sensitive strains equally. The annual incidence rate ratio ( *IRR*) associated with the intervention was calculated for hospital- and community-acquired infections of both strains.

**Results**: For the resistant strain, there were large reductions in hospital-acquired infections (0.1 ≤ *IRR* ≤ 0.6) and smaller reductions in community-acquired infections (0.2 ≤ *IRR* ≤  0.9). These reductions increased in line with increasing importance of nosocomial transmission of the strain. For the sensitive strain, reductions in hospital acquisitions were much smaller (0.6 ≤ *IRR* ≤ 0.9), while communityacquisitions could increase or decrease (0.9 ≤ *IRR* ≤ 1.2). The greater the importance of the community environment for the transmission of the sensitive strain, the smaller the reductions.

**Conclusions**: Counter-intuitively, infection control interventions, including hand hygiene, can have strikingly discordant effects on resistant and sensitive strains even though they target them equally, following differences in their adaptation to hospital and community-based transmission. Observed lack of effectiveness of control measures for sensitive strains does not provide evidence that infection control interventions have been ineffective in reducing resistant strains.

## Introduction

In England and Wales, rates of methicillin-resistant *Staphylococcus aureus* (MRSA) bacteraemia in hospitals showed a sharp decline following implementation of the national CleanYourHands campaign in 2004, with rates falling from 1.9 to 0.9 cases per 10 000 bed days between 2004 and 2008 ^1^. Over the same period, the methicillin-sensitive *Staphylococcus aureus* (MSSA) bacteraemia rate showed a small increase from 2.7 per 10 000 bed days in 2004 to 3.0 in 2008. Analysis of regional or hospital-level data from England reveals a similar picture: most hospital settings experienced sharp falls in rates of MRSA infection from 2004, while MSSA infection rates either did not fall or fell only in line with pre-existing trends ^2, 3^. A remarkably similar pattern has recently been reported for *Clostridium difficile* infection (CDI) in England ^4^. CDI prevention policies, including infection control and antibiotic stewardship, were introduced in England in 2007; by 2013 the annual number of CDI had fallen by approximately 80 per cent. Genomic analysis revealed that this decline was accounted for by the elimination of fluoroquinolone-resistant strains. Rates of infection with fluoroquinolone-sensitive strains showed very little change following the interventions, and there was no change in the number of inferred secondary cases with or without hospital contact.

These diverging outcomes for antibiotic-resistant and antibiotic-sensitive variants of common nosocomial pathogens have led some researchers to argue that these data provide evidence against infection control measures having played a major role in these declines ^3, 4^. It is reasoned that non-specific infection control measures, such as improved hand hygiene or ward cleaning, would be expected to reduce hospital transmission of resistant and sensitive strains equally. The fact that we observe only declines in resistant strains indicates that other factors, i.e. those having a differential effect on resistant and sensitive strains, must have been the major causes for the reduction ^4^. Here we develop a simple mechanistic mathematical model to assess the validity of this line of reasoning. Our model considers the carriage dynamics of two bacterial strains: one antibiotic-resistant and one antibiotic-sensitive. We assume that both strains are able to spread between individuals in the hospital and the community, but that the resistant strain transmits better in the hospital, while the sensitive strain transmits better in the community.

Since most bacterial hospital pathogens of clinical concern can be carried asymptomatically over long periods, we account for movements of colonized individuals between the hospital and community ^5^. We explicitly model a hospital hand hygiene intervention as an example of a non-specific infection control measure and evaluate the impact of this intervention on the incidence of hospital and community acquisitions of antibiotic-resistant and antibiotic-sensitive strains.

## Methods

### Model framework and assumptions

**Figure 1.**
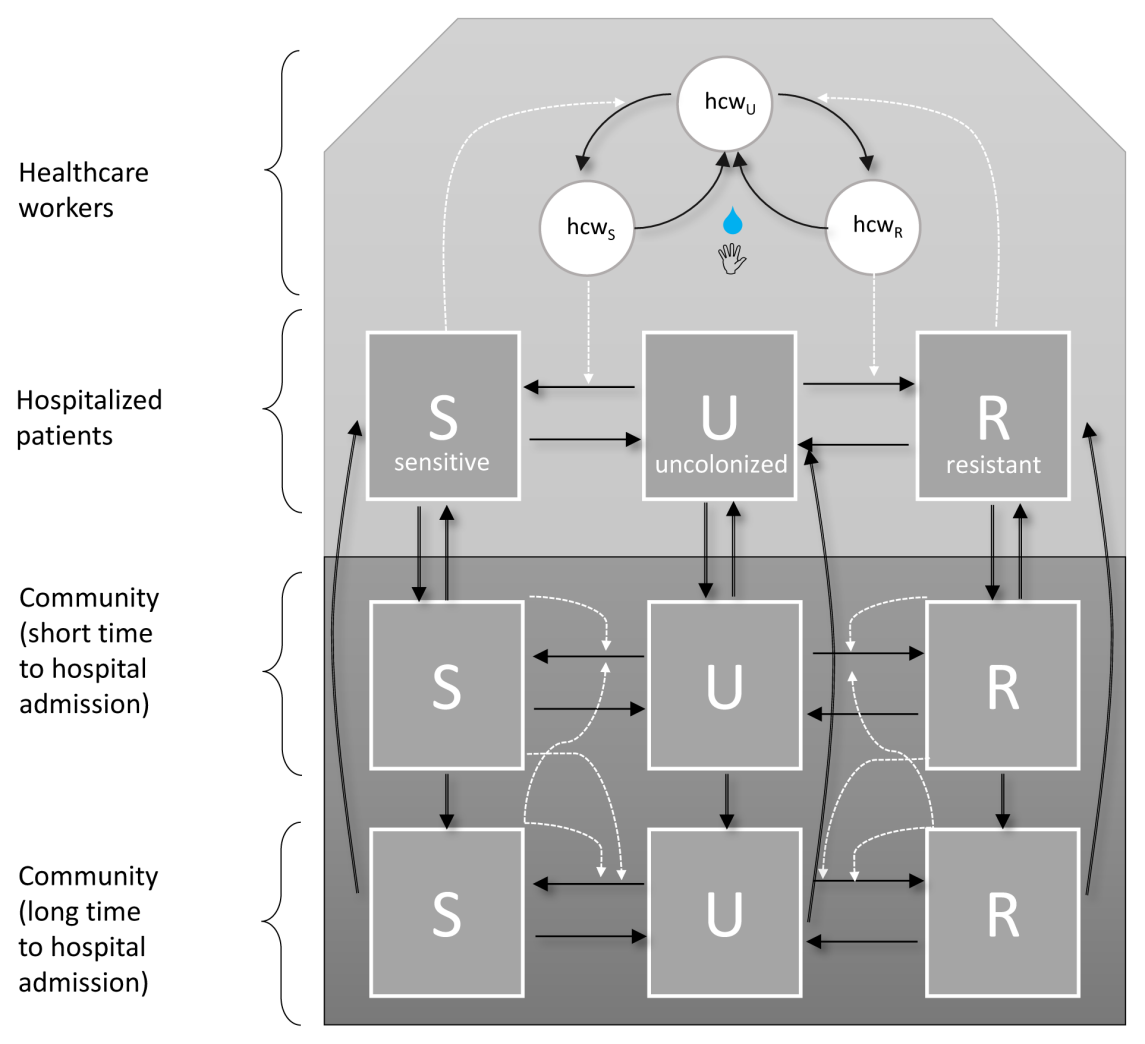
Flow diagram of model framework. In all three populations, individuals can reside in and move between the three carriage states (uncolonized, colonized with antibiotic-sensitive bacteria, and colonized with antibiotic-resistant bacteria). Movements between states are indicated by black arrows. Broken white lines indicate what variables influence transition rates between compartments. Transmission events between hospitalized patients are mediated by transiently contaminated healthcare workers (circles), and transient contamination is removed by hand hygiene events (an intervention which affects resistant and sensitive strains equally).

We developed a dynamic deterministic compartmental transmission model to track the number of people colonized with the resistant and sensitive strains in the hospital and community ( Figure 1).

Transmission between patients in the hospital was assumed to occur via the transiently contaminated hands of healthcare workers. We modelled this process explicitly using a previously described host-vector approach ^6, 7^. Persistent carriage of bacteria such as MRSA has been reported among healthcare workers, though is commonly found to be transient ^8^. Therefore, healthcare workers in turn were assumed to become transiently contaminated through patient contact. Hand hygiene performed by a contaminated healthcare worker was assumed to clear this contamination ^9^. Individuals were considered to be either colonized with an antibiotic-sensitive strain (whether asymptomatically or symptomatically), colonized with an antibiotic-resistant strain or uncolonized and susceptible to both.

Patients were tracked by their hospitalisation history so that recently discharged patients (those in population 2) experienced a transient period with a shorter expected time to their next hospital admission; i.e. a higher (re)admission rate than the general community population (population 3, Figure 1). We assumed frequency-dependent transmission ^10^. The model allowed for the possibility of assortative mixing within populations 2 and 3, where the effective contact rate of strain *i* ( *β _i _n__*) between individuals within a population *n* was a fraction of the effective contact rate between individuals across populations:

$\begin{array}{l} \begin{array}{ccc} & \;N_{2} & \;N_{3} \end{array}\\ \begin{array}{c} N_{2}\\ N_{3} \end{array}\left(\begin{array}{cc} \beta_{R_{2}} & f_{23}\beta_{R_{3}}\\ f_{32}\beta_{R_{2}} & \beta_{R_{3}} \end{array}\right) \end{array}$

The resistant strain was assumed to be better adapted to the hospital setting, meaning that in the absence of other colonized hosts, a patient colonized with a resistant strain admitted to the hospital would be expected to generate more secondary cases during their hospital episode than a patient colonized with a sensitive strain. In contrast, the sensitive strain was assumed to be better adapted to the community. Individuals could not be co-infected with resistant and sensitive strains, and we allowed for bacterial interference between the two strains so that colonization with one strain reduced the risk of acquisition of the other strain, with at baseline assuming 100% bacterial interference, i.e. no replacement infection ^11, 12^.

Model equations are given below. Variables are defined in Table 1 and parameter definitions and values in Table 2.

**Table 1.** Model variables.

Variable	Description
*U*_1_	Susceptible population 1: number of patients in hospital who are not colonized/infected with either the resistant or sensitive strain.
*U*_2_	Susceptible population 2: number of individuals in community setting who have a short expected time to hospital admission who are colonized with neither the resistant nor the sensitive strain.
*U*_3_	Susceptible population 3: number of individuals in community setting who have a long expected time to hospital admission who are colonized with neither the resistant nor the sensitive strain
*R*_1_	Resistant population 1: number of patients in hospital setting colonized with the resistant (hospital-adapted) strain
*R*_2_	Resistant population 2: number of individuals in community setting who have a short expected time to hospital admission who colonized with the resistant (hospital-adapted) strain
*R*_3_	Resistant population 3: number of individuals in community setting who have a long expected time to hospital admission who colonized with the resistant (hospital-adapted) strain
*S*_1_	Sensitive population 1: number of patients in hospital setting colonized with the sensitive (community-adapted) strain
*S*_2_	Sensitive population 2: number of individuals in community setting who have a short expected time to hospital admission who colonized with the sensitive (community-adapted) strain
*S*_3_	Sensitive population 3: number of individuals in community setting who have a long expected time to hospital admission who colonized with the sensitive (community-adapted) strain
*hcw _R_*	Number of hospital healthcare workers who are transiently colonized with the resistant (hospital-adapted) strain
*hcw _S_*	Number of hospital healthcare workers who are transiently colonized with the sensitive (community-adapted) strain

**Table 2.** Model parameters. ^*^Defined by other parameters to give *R* values of 1.5 for the resistant and 1.4 for the sensitive strain. Here *R _n_* is defined as the expected number of secondary cases in the hospital and community resulting from one colonised individual in a fully uncolonized and susceptible population at baseline hand hygiene rates of 40%, accounting for the possibility of readmissions while still colonized.

Parameter	Description	Value (range)	Source
*N*_1_	Number of hospitalised patients	1000	
*N _hcw_*	Number of healthcare workers (HCW)	100	13, 14
*N*_2_	Number of people in the community who have a short expected time to hospital admission (recently discharged people)	10,000	
*N*_3_	Number of people in the community who have a long expected time to hospital admission (not recently discharged people)	100,000	
*τ*	Hospital patient removal rate (reciprocal of mean hospital stay)	1/10 *d* ^−1^	
*ν*	Rate of transition from the community population with a high hospital admission rate to the community population with a low hospital admission rate (reciprocal of mean duration with a high admission rate)	*hN*_3_ */N*_2_	
*ρ*	Ratio of hospital admission rate of the recently hospitalised to hospital admission rate for the general population	20	
*h*	Admission rate to hospital of people in the general population	See methods	
*r*	Admission rate to hospital of recently discharged people	See methods	
*c*	Mean number of HCW contacts per patient day	10	
*γ*_*R*_1__, *γ*_*R*_2__, *γ*_*R*_3__	Carriage clearance rate of the resistant (hospital-adapted) strain in the hospital/community (reciprocal of mean carriage duration)	1/400 *d* ^−1^	15
*γ*_*S*_1__, *γ*_*S*_2__, *γ*_*S*_3__	As above for the sensitive (community-adapted) strain	1/40 *d* ^−1^,1/400 *d* ^−1^,1/400 *d* ^−1^	
*β*_*R*_1__	Transmission parameter for the resistant strain (from colonized HCW to a susceptible patient)	0.187 (0.035,0.225)	*
*β*_*S*_1__	As above for the sensitive strain	0.100 (0.040,0.216)	*
*p*	Ratio of probability of transmission from colonized patient to a susceptible HCW to the probability of transmission from colonized HCW to a susceptible patient	10	
*β*_*R*_2__, *β*_*R*_3__	Transmission parameters for the resistant strain in the community populations	0.00212 (0.00013,0.00335)	*
*β*_*S*_2__, *β*_*S*_3__	As above for the sensitive strain	0.00320 (0.00236,0.00330)	*
*λ*_*R*_1__, *λ*_*R*_2__, *λ*_*R*_3__	Rate at which uncolonized individuals become infected with the resistant strain per unit time in the hospital/community	See methods	
*λ*_*S*_1__, *λ*_*S*_2__, *λ*_*S*_3__	As above for the sensitive strain	See methods	
*H*	Baseline hand hygiene compliance (probability of successful hand decontamination following patient contact)	40%	
*η*	Hand hygiene rate	See methods	
*ω _R_*	Bacterial interference: risk ratio for acquiring the resistant strain if carrying the sensitive strain relative to a non-carrier	0 (0, 1)	
*ω _S_*	As above for the sensitive strain	0 (0, 1)	
*f*_23_	The ratio of the effective contact rate in *N*_2_ from someone in *N*_3_ to the effective contact rate in *N*_3_ from someone in *N*_3_ (where 1 implies that on contact, someone in *N*_3_ is causing new infections in *N*_3_ and *N*_2_ at the same rate). Of note, as *N*_3_ > *N*_2_, *f*_23_ = *N*_2_/ *N*_3_ assumes the same per capita infection rate, i.e. homogeneous mixing.	*N*_2_/ *N*_3_ (0, *N*_2_/ *N*_3_)	
*f*_32_	The ratio of the effective contact rate in *N*_3_ from someone in *N*_2_ to the effective contact rate in *N*_2_ from someone in *N*_2_.	1 (0, *N*_3_/ *N*_2_)	
-	Percentage of transmission events with the hospital-adapted strain (assuming an otherwise fully susceptible population, and that the hospital- adapted strain is initially acquired in the hospital)	25% (0%, 60%)	
-	As above for the community-adapted strain	2.5% (0%, 15%)	

## Equations

$\frac{dU_{1}}{dt}$ = *rU*_2_ + *hU*_3_ – *τU*_1_ – *λ*_*R*_1__ *U*_1_ – *λ*_*S*_1__ *U*_1_ + *γ*_*R*_1__ *R*_1_ + *γ*_*S*_1__ *S*_1_                           (1)

$\frac{dU_{2}}{dt}$ = – *rU*_2_ – *νU*_2_ + *τU*_1_ – *λ*_*R*_2__ *U*_2_ – *λ*_*S*_2__ *U*_2_ + *γ*_*R*_2__ *R*_2_ + *γ*_*S*_2__ *S*_2_                         (2)

$\frac{dU_{3}}{dt}$ = – *hU*_3_ + *νU*_2_ – *λ*_*R*_3__ *U*_3_ – *λ*_*S*_3__ *U*_3_ + *γ*_*R*_3__ *R*_3_ + *γ*_*S*_3__ *S*_3_                                   (3)

$\frac{dR_{1}}{dt}$ = *rR*_2_ + *hR*_3_ – *τ* *R*_1_ – *γ*_*R*_1__ *R*_1_ + *λ*_*R*_1__ *U*_1_ + *ω*_*R*_ *λ*_*R*_1__ *S*_1_ – *ω*_*S*_ *λ*_*S*_1__ *R*_1_                   (4)

$\frac{dR_{2}}{dt}$ = – *rR*_2_ – *νR*_2_ + *τ* *R*_1_ – *γ*_*R*_2__ *R*_2_ + *λ*_*R*_2__ *U*_2_ + *ω*_*R*_ *λ*_*R*_2__ *S*_2_ – *ω*_*S*_ *λ*_*S*_2__ *R*_2_                 (5)

$\frac{dR_{3}}{dt}$ = – *hR*_3_ + *vR*_2_ – *γ*_*R*_3__ *R*_3_ + *λ*_*R*_3__ *U*_3_ + *ω*_*R*_ *λ*_*R*_3__ *S*_3_ – *ω*_S_ *λ*_*S*_3__ *R*_3_                         (6)

$\frac{dS_{1}}{dt}$ = *rS*_2_ + *hS*_3_ – *τS*_1_ – *γ*_*S*_1__ *S*_1_ + *λ*_*S*_1__ *U*_1_ + *ω*_*S*_ *λ*_*S*_1__ *R*_1_ – *ω*_*R*_ *λ*_*R*_1__ *S*_1_                    (7)

$\frac{dS_{2}}{dt}$ = – *rS*_2_ – *νS*_2_ + *τS*_1_ – *γ*_*S*_2__ *S*2 + *λ*_*S*_2__ *U*_2_ + *ω _S_* *λ*_*S*_2__ *R*_2_ – *ω _R_* *λ*_*R*_2__ *S*_2_                   (8)

$\frac{dS_{3}}{dt}$ = – *hS*_3_ + *vS*_2_ – *γ*_*S*_3__ *S*_3_ + *λ*_*S*_3__ *U*_3_ + *ω*_*S*_ *λ*_*S*_3__ *R*_3_ – *ω*_*R*_ *λ*_*R*_3__ *S*_3_                             (9)

$\frac{dhcw_{R}}{dt}$ = *p* *β*_*R*_1__ *R*_1_( *N _hcw_* – *hcw _R_* – *hcw _S_*)/ *N _hcw_* – *ηhcw _R_*                                   (10)

$\frac{dhcw_{S}}{dt}$ = *p* *β*_*S*_1__ *S*_1_( *N _hcw_* – *hcw _R_* – *hcw _S_*)/ *N _hcw_* – *ηhcw _S_*                                     (11)

*h* = *τ* *N*_1_⁄( *N*_3_ + *ρ* *N*_2_)

*r* = *ρ* *h*

*λ*_*R*_1__ = *β*_*R*_1__ *hcw _R_*⁄ *N _hcw_*

*λ*_*R*_2__ = *β*_*R*_2__ *R*_2_⁄ *N*_2_ + *β*_*R*_3__ *R*_3_ *f*_23_⁄ *N*_2_

*λ*_*R*_3__ = *β*_*R*_3__ *R*_3_⁄ *N*_3_ + *β*_*R*_2__ *R*_2_ *f*_32_⁄ *N*_3_

*λ*_*S*_1__ = *β*_*S*_1__ *hcw _S_*⁄ *N _hcw_*

*λ*_*S*_2__ = *β*_*S*_2__ *S*_2_⁄ *N*_2_ + *β*_*S*_3__ *S*_3_ *f*_23_⁄ *N*_2_

*λ*_*S*_3__ = *β*_*S*_3__ *S*_3_⁄ *N*_3_ + *β*_*S*_2__ *S*_2_ *f*_32_⁄ *N*_3_

*η* = *HcN*_1_⁄( *N _hcw_*(1 – *H*))

*N*_1_ = *U*_1_ + *R*_1_ + *S*_1_

*N*_2_ = *U*_2_ + *R*_2_ + *S*_2_

*N*_3_ = *U*_3_ + *R*_3_ + *S*_3_

The net reproduction numbers ( *R*) for both resistant and sensitive pathogens (1.5 and 1.4, respectively) were calculated as the dominant eigenvalues of the next generation matrix ^16^. Here, *R* is defined as the expected number of secondary cases in the hospital and community resulting from one infected individual in a fully uncolonized and susceptible population at baseline hand hygiene rates of 40%, accounting for the possibility of readmissions while still colonized. The model was implemented by numerically solving the set of ordinary differential equations using R version 3.3.1 (Team R Development Core, website: https://cran.r-project.org/) and the package deSolve ^17^. Model code is available online ^18^.

### Hospital infection control measures

We modelled a hospital infection control intervention to reduce secondary spread of bacterial pathogens in the hospital. This was achieved by a stepwise increase in hand hygiene compliance amongst health care workers from a baseline rate of 40% to a rate of 50%. We assumed the intervention was equally effective at decontaminating hands of healthcare workers transiently contaminated with resistant and sensitive strains.

### Measuring the impact of hospital infection control

Annual incidence rate ratios (IRR) were calculated using simulated data for one year pre- and post-intervention ( *T*_0_ and *T*_1_ respectively) after first running the model to equilibrium. To aid comparison with reported infection data ^4^, we assumed the number of new infections with and without a hospital link ( *Y _i _n__*) was proportional to the cumulative number of acquisitions (Δ *I _i _n__*) in the hospital or community, respectively, in each of the two time periods:

$\begin{array}{l} \Delta I_{S_{1}}\left(t\right)=\int_{T_{i}}^{T_{i}+365}\lambda_{S_{1}}U_{1}\text{d}t\\ \Delta I_{R_{1}}\left(t\right)=\int_{T_{i}}^{T_{i}+365}\lambda_{R_{1}}U_{1}\text{d}t\\ \Delta I_{S_{23}}\left(t\right)=\int_{T_{i}}^{T_{i}+365}\lambda_{S_{2}}U_{2}+\lambda_{S_{3}}U_{3}\text{d}t\\ \Delta I_{R_{23}}\left(t\right)=\int_{T_{i}}^{T_{i}+365}\lambda_{R_{2}}U_{2}+\lambda_{R_{3}}U_{3}\text{d}t \end{array}$

Confidence intervals were calculated using 1000 Monte Carlo replicates on the assumption that the actual number of observed infections of each strain ( *Y _i _n__*) followed a negative binomial distribution where *Var*( *Y _i _n__*) = *μ* + *μ* ^2^/ *κ*, with *κ* (the dispersion parameter) = *μ*/( *θ* − 1), with *θ* = 5, and assuming 1 in 10 carriage episodes acquired in hospital resulted in a reported infection. This was 1 in 50 for community-acquired episodes. Hence we allowed for differences in reporting rates as well as heterogeneity in case-mix between both settings, affecting the likelihood of developing an infection. Then the *IRR _i _n__* corresponded to the ratio of the number of new observed infections of strain *i* in population *n* in the year pre-intervention to the number in the first year post-intervention:

$IRR_{i_{n}}=\frac{\sum_{t=T_{1}}^{T_{1}+365}Y_{i_{n}(t)}}{\sum_{t=T_{0}}^{T_{0}+365}Y_{i_{n}(t)}}$

### Investigating the importance of environmental adaptation of competing pathogens

At baseline, the relative fraction of new cases acquired in hospital was 25% and 2.5% for the resistant and sensitive strains, respectively. To investigate the impact of hospital- and community-adaptation of both strains on our findings, we varied the level of transmission in both settings for each of the two strains, while keeping the overall net reproduction number for resistant and sensitive strains constant at 1.5 and 1.4, respectively. We investigated hospital acquisition fractions of 0.5–60%, for the resistant strain, and 0.5–15% for the sensitive strain. Only scenarios where resistant and sensitive strains co-existed prior to the intervention were considered in this analysis, and we considered this to be the case when the equilibrium incidence rates for colonization were above one per 100,000 person years for both strains.

## Results

### Impact of hospital infection control

**Figure 2.**
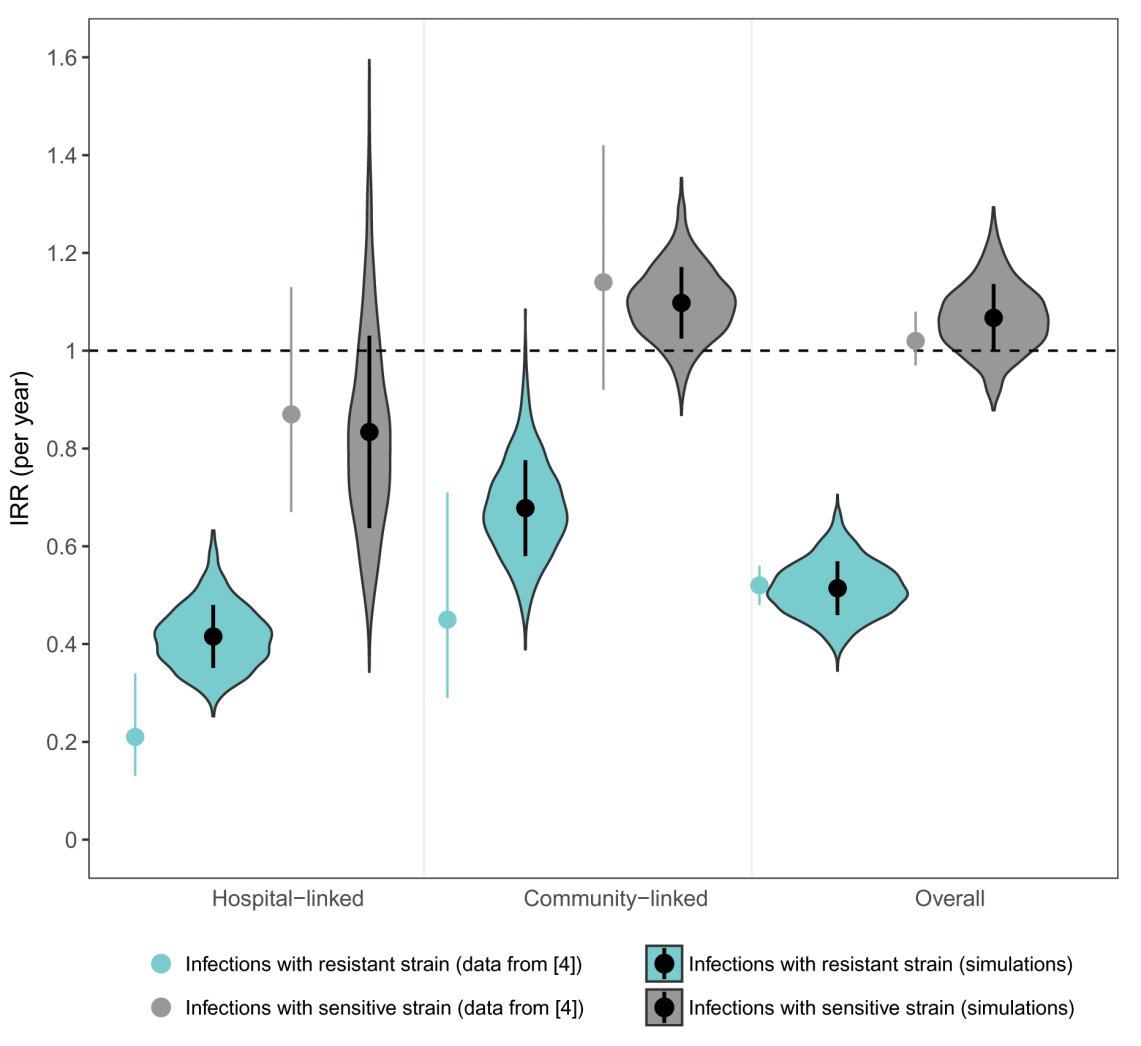
Distribution of predicted incidence rate ratios associated with the infection control intervention. Predicted annual incidence rate ratios (IRRs) for infections with the resistant and sensitive bacterial strains associated with a 10% improvement in hand hygiene compliance from a baseline of 40%. Incidence rate ratios were calculated using simulated data one year pre- and post-intervention, where observed infections followed a negative binomial distribution with a mean proportional to the number of acquisitions in hospital and community in the deterministic model. Shaded areas represent distributions, and enclosed dots and lines represent medians and standard deviations. An IRR of 1 corresponds to no change (dotted line). Non-enclosed single dots and lines represent mean and 95% confidence intervals of observed IRRs for *C. difficile* fluoroquinolone-resistant (turquoise) and fluoroquinolone-sensitive (grey) strains, grouped according to presence or absence of a hospital link (data from ^4^).

Improving hand hygiene compliance by 10% resulted in dramatic reductions in the incidence of infections with the resistant strain. These reductions were most pronounced for secondary cases that resulted from cross-infection within the hospital (IRR = 0.41 [95% CI: 0.32–0.52] under baseline parameters); they were also clearly observed for acquisitions that occurred in the community (IRR = 0.67 [0.59–0.76], Figure 2). Incidence rates of infections caused by the sensitive strain were markedly less affected by the intervention, though in the first year post-intervention there was a moderate reduction in infections linked to hospital transmission (IRR = 0.83 [0.55–1.22] Figure 2). In contrast, the reduced competition from the resistant strain resulted in moderate increases in sensitive infections linked to community acquisitions (IRR = 1.10 [1.03–1.17], Figure 2). The net result was a small overall increase in the incidence of infections with the sensitive pathogen. These trends are exactly in line with reported data ^4^ ( Figure 2).

### Dynamics after hospital infection control

**Figure 3.**
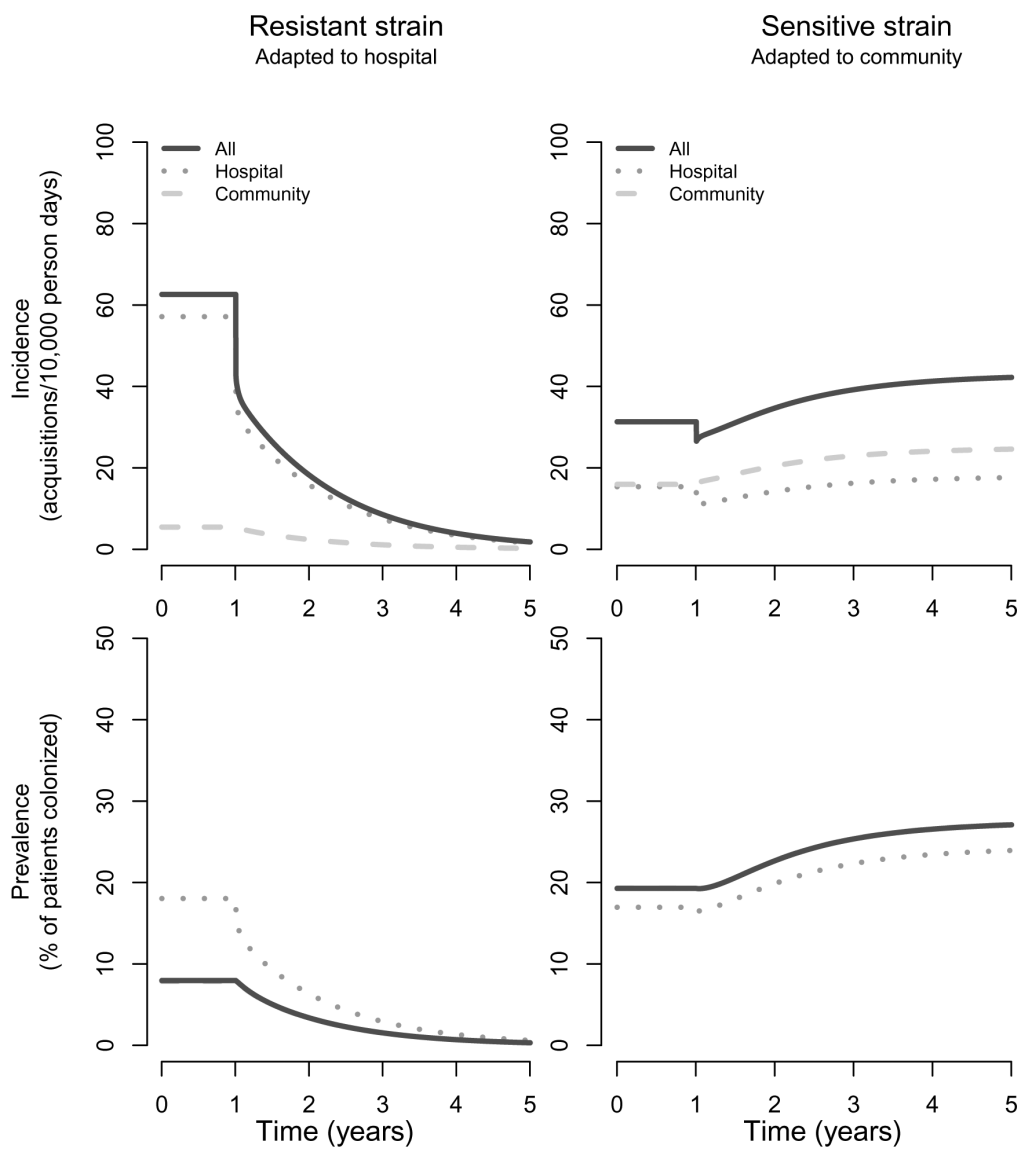
Predicted incidence and prevalence trends of the sensitive and resistant bacterial strains following the introduction of enhanced infection control. Trends in the incidence of new acquisitions (symptomatic and asymptomatic) and carriage prevalence for resistant and sensitive bacterial strains following a 10% stepwise improvement in hand hygiene compliance after one year from a baseline of 40%. Incidence trends are depicted as transmission events following from community-to-community transmission (dashed line) and hospital-to-hospital transmission (dotted line). As prevalence in the hospital represents only a small fraction of the overall prevalence (in hospital and community populations combined), the latter is almost identical to the community prevalence for both the resistant and sensitive bacterial strains.

The above results appear counterintuitive, but can be understood after consideration of the dynamics. First, the reduction in resistant infections linked to community transmission can be explained by a reduction in the number of patients colonized with resistant bacteria at hospital discharge. Reducing the efflux of these colonized patients into the community (a consequence of reduced transmission in the hospital) leads to a long-term decline in the prevalence and incidence of the resistant strain in this setting ( Figure 3). These gradual changes in the community reservoir (which occur despite the sudden changes in the hospital transmission rate due to the intervention) in turn lead to reduced importations (and subsequent transmission) of the resistant strain into the hospital. This explains why we see a gradual decline in resistant infections in the hospital and community even following an intervention that occurs in a stepwise manner and which is restricted to the hospital.

For the sensitive pathogen strain, we also see an initial stepwise reduction in the hospital incidence of new patient acquisitions ( Figure 3). However, the drop is smaller than for the resistant strain because the sensitive strain depends much less on hospital transmission for maintaining its hospital prevalence and much more on importations from the community. Despite this initial fall in hospital prevalence and incidence of the sensitive strain, over a period of several years there are modest increases in both - a consequence of reduced competition with the resistant strain. The net result is that the intervention has a discordant effect on new hospital acquisitions of the sensitive and resistance strains; the former marginally increases over a period of several years, while the latter declines to low levels.

Broadly similar dynamics were observed for larger increases in hand hygiene compliance, and for sufficiently high compliance the intervention was capable of driving the resistant strain to extinction ( Supplementary Figure S1 and Supplementary Figure S2). Thus, while the resistant strain was able to persist at a low level alongside the sensitive strain when hand hygiene compliance was 50% ( Figure 3), increasing it further to >55% induced a more rapid decline in both the hospital and community reservoir and successfully eliminated the resistant strain within the five year time period simulated ( Supplementary Figure S1 and Supplementary Figure S2).

### Importance of the degree of strain adaptation to the hospital and community settings

**Figure 4.**
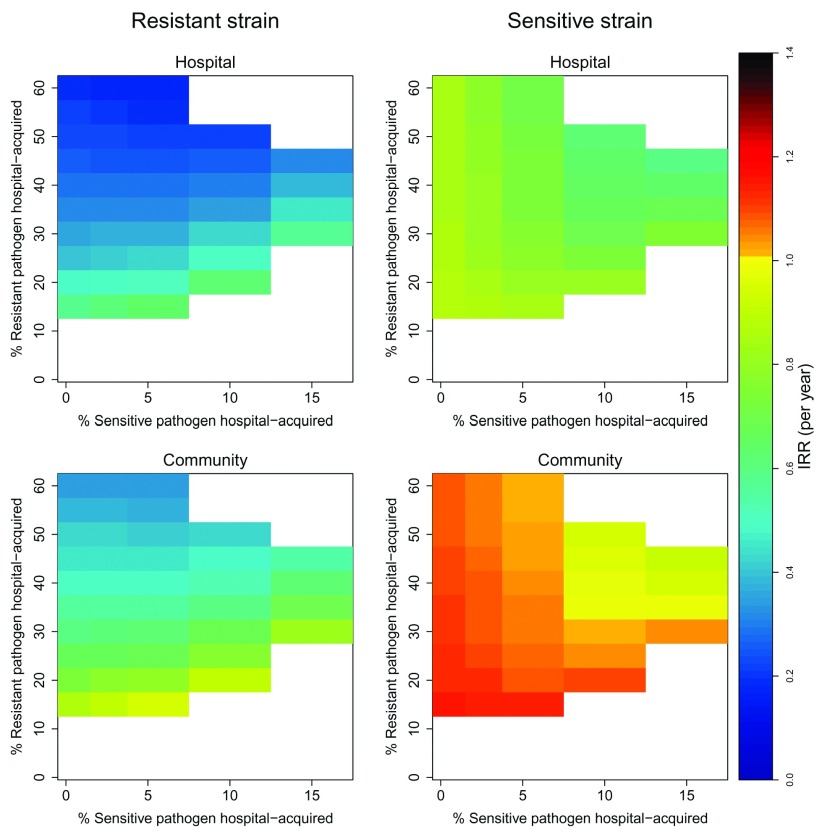
Annual incidence rate ratios of new acquisitions (symptomatic and asymptomatic) associated with an infection control intervention under different levels of adaptation of sensitive and resistant strains to hospital and community settings. In all simulations, reproduction numbers for resistant and sensitive strains were held constant at 1.5 and 1.4, respectively. For corresponding transmission parameter values, see https://github.com/esthervankleef/Two_strain_model_published/tree/v1.0.3. White spaces represent scenarios where no co-existence occurred. An IRR = 1 corresponds to no change.

With baseline parameters, 25% of acquisitions of the resistant strain occurred in hospital; the corresponding figure for the sensitive strain was 2.5%. Increasing adaptation of the resistant strain to the hospital environment (i.e. increasing the proportion of resistant transmission that occurs in hospital by changing the values of the transmission parameters ( *β _i _n__*) while keeping the net reproduction number and all other parameters constant), resulted in larger effect sizes for the hospital infection control intervention: 0.1 ≤ *IRR* ≤ 0.6 for incidence linked to hospital transmission and 0.2 ≤ *IRR* ≤ 0.9 for incidence related to community transmission ( Figure 4). For the sensitive strain, secondary cases with a hospital link also declined in response to the intervention, though at lower rates than the resistant strain (0.6 ≤ *IRR* ≤ 0.9).

In contrast, incidence rates of the sensitive pathogen without a hospital link either remained unchanged or increased following the infection control intervention(0.9 ≤ *IRR* ≤ 1.2). The smaller the importance of the hospital environment for transmission of the sensitive strain, the larger the increase in its incidence rate in the community in response to the intervention ( Figure 4). This increase became larger when the percentage of resistance strain acquisitions occurring in the community increased.

## Discussion

Our analysis shows that discordant temporal changes in resistant and sensitive infections in response to intensified hospital-based control measures, as observed for *Staphylococcus aureus* ^2, 3^ and *C. difficile* ^4^, are consistent with an intervention that reduces transmission rates of resistant and sensitive bacteria equally. Under plausible assumptions (all of which have been used in previous models ^5, 6, 19, 20^) our simulations were able to produce effect sizes that are similar to those observed with real data ^4^. Notably, we did not assume the existence of an intervention, such as antimicrobial stewardship, that has different direct effects on resistant and sensitive strains. Some aspects of our results (and of the real-world data) may be considered counterintuitive ^3, 4^, but the modelling framework helps provide a simple intuitive explanation. In general, if two pathogen strains compete unequally in two environments, a transmission-reducing intervention that preferentially targets one environment will have a disproportionate effect on the strain better adapted to that environment. We have used a hand hygiene intervention as our motivating example; similar conclusions would have been reached with other non-specific hospital infection control measures, such as ward cleaning.

Previous modelling work has shown that hospital infection control measures can have a greater effect on resistant than on sensitive bacteria ^19^. This can be expected when the hospital influx of patients carrying sensitive bacteria is the dominant factor in maintaining their high hospital prevalence, while patient-to-patient spread is largely responsible for the high hospital prevalence of resistant bacteria. Our model has extended this work by explicitly accounting for transmission in the community reservoir. One motivation for doing this is to allow direct comparison with data from recent studies using whole genome sequencing to identify infections plausibly linked to recent hospital transmission ^4^. Consideration of hospital and community dynamics also enabled us to capture the observed long-term temporal changes in resistance in response to interventions, and to demonstrate that the prevalence of sensitive bacteria may in fact marginally increase following non-specific infection control measures. We have not attempted to quantify the relative contributions of infection control, antibiotic stewardship and other factors in the large reductions in nosocomial infections with *C. difficile* and *S. aureus* in England and Wales. Our analysis merely shows that the observed reductions in resistant infections without reductions in sensitive infections is not inconsistent with infection control playing a major role. There are other lines of evidence to suggest infection control may have made an important contribution. For example, in England and Wales strong negative associations between hospital-level usage of soap and *C. difficile* infection rates and between alcohol hand rub and MRSA infection rates have been reported ^1^. Similar associations have been reported elsewhere (e.g. Vernaz *et al.*, 2009 ^21^).

The intensification of hospital infection control is commonly multifaceted, complicating the quantification of the effectiveness of individual interventions. Our findings indicate further data, e.g. hospital-level antimicrobial consumption data and measures of the behavioural impact of infection control interventions, are required to more reliably quantify the relative contribution of different control measures to the reductions observed. The most detailed analysis to date comes from two long time series studies from northeast Scotland ^22, 23^. These suggest that both antibiotic stewardship and infection control measures made important contributions to the decline in MRSA infections in this region, while an antibiotic stewardship intervention (restricting the use of fluoroquinolones, clindamycin, co-amoxiclav, and cephalosporins) was likely to have been the dominant factor in reducing *C. difficile* infections. A strong point of our work is the simple framework we used for considering generic pathogens. The flexibility of the model readily allows adaptation to specific pathogens. For example, assumptions about carriage duration, mixing of community populations, and the degree of bacterial interference between the two strains can easily be altered (and will not change our main conclusions, as shown in respectively ^24^, Figure S3, Figure S4)”. In addition, by capturing dynamic transmission in both hospital- and community-populations (something commonly ignored in mathematical models of nosocomial pathogens ^25^), and including a *core group* of recently discharged patients with higher readmission rates, we were able to capture the interaction between hospital and community more realistically. Of note, this core group is not an essential model requirement for our central result, which is that infection control interventions alone can account for the very different effects on sensitive and resistant strains.

Our work also has important limitations. All models are simplifications of reality. Hospitals and communities encompass complex networks of contact patterns; our model represents only a caricature of these networks. We did not allow for co-infection with resistant and sensitive strains. This is a reasonable approximation for *S. aureus* ^11^, and competition for ecological niches has been reported for *C. difficile* (e.g. Songer *et al.*, 2007 ^26^; Merrigan *et al.*, 2003 ^27^), but it is unclear how appropriate this assumption would be for other enteric pathogens. Clearly, our model also ignores a lot of host and pathogen heterogeneity, nor did we account for stochastic effects. In small populations of single hospitals, chance events are likely to play an important role in the transmission dynamics of pathogens. Moreover, for simplicity we chose to focus on what appears to be the dominant mode of transmission (at least for *S. aureus*, other organisms are less well studied). Since hospitalised patients are generally not mobile, patient-to-patient transmission events represent either hand-borne or air-borne transmission. Studies from the 1960s suggest that the latter plays a relatively minor role in *S. aureus* transmission in hospital settings ^28, 29^. However, we can think of no plausible mechanism by which incorporation of more biological realism would in any way alter our primary conclusion. Though our framework allows for further complexity, the purpose here was to demonstrate that the divergent effects of infection control interventions on resistant and sensitive models could be explained even with a simple model. Therefore, no formal model fitting to data was conducted. However, we have presented a set of scenarios for different degrees of hospital-adaptation, making our findings generalizable to a wide variety of settings and pathogens.

## Conclusions

Hospital-based infection control interventions, such as hand hygiene, that target sensitive and resistant bacteria equally, can result in diverging outcomes for strains which are differentially adapted to community and hospital transmission. While it is highly plausible that changing patterns of antibiotic usage have played an important role in some of the observed declines in *C. difficile* and *S. aureus* infections, the relative importance of antibiotic stewardship versus infection control interventions cannot be inferred from differential changes in infection rates with resistant and sensitive bacteria.

## Software availability

Latest source code: https://github.com/esthervankleef/Two_strain_model_published/tree/v1.0.3 ^18^

Archived source code as at the time of publication: http://doi.org/10.5281/zenodo.1045530 ^18^

License: MIT license

## Supplementary material

**Figure S1: Predicted trends in incidence of new acquisitions of sensitive and resistant strains under varying improvements in hand hygiene compliance.** Trends in the incidence of new acquisitions (symptomatic and asymptomatic) for resistant and sensitive bacterial strains following a 5%, 10%, 15% and 20% improvement respectively in hand hygiene compliance from a baseline of 40%.

**Figure S2: Predicted trends in prevalence of new acquisitions of sensitive and resistant strains under varying improvements in hand hygiene compliance.** Trends in the prevalence of carriage (symptomatic and asymptomatic) for resistant and sensitive bacterial strains following a 5%, 10%, 15% and 20% improvement respectively in hand hygiene compliance from a baseline of 40%.

**Figure S3: Predicted trends in incidence of new acquisitions of sensitive and resistant strains under varying degrees of bacterial interference.** Trends in the incidence of new acquisitions (symptomatic and asymptomatic) for resistant and sensitive bacterial strains following 10% improvement respectively in hand hygiene compliance from a baseline of 40%.

**Figure S4: Predicted trends in incidence of new acquisitions of sensitive and resistant strains under varying degrees of social mixing between community populations.** Trends in the incidence of new acquisitions (symptomatic and asymptomatic) for resistant and sensitive bacterial strains following 10% improvement respectively in hand hygiene compliance from a baseline of 40%.
