# Supplementary figures and images for: Why sensitive bacteria are resistant to hospital infection control
Source: Wellcome Open Res. 2017 Nov 22;2:16. Originally published 2017 Mar 10. [Version 2] doi: 10.12688/wellcomeopenres.11033.2 (PMC5721567; doi:10.12688/wellcomeopenres.11033.2)

## Resistant strain

Adapted to hospital

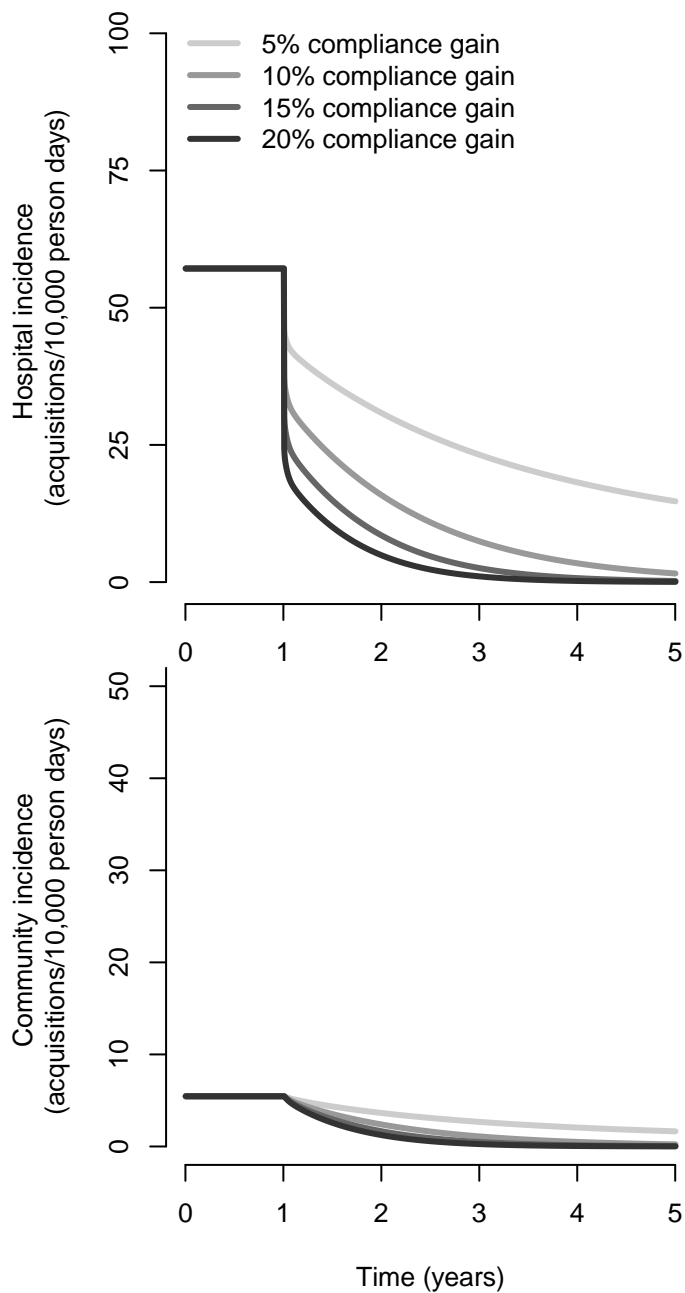

## Sensitive strain

Adapted to community

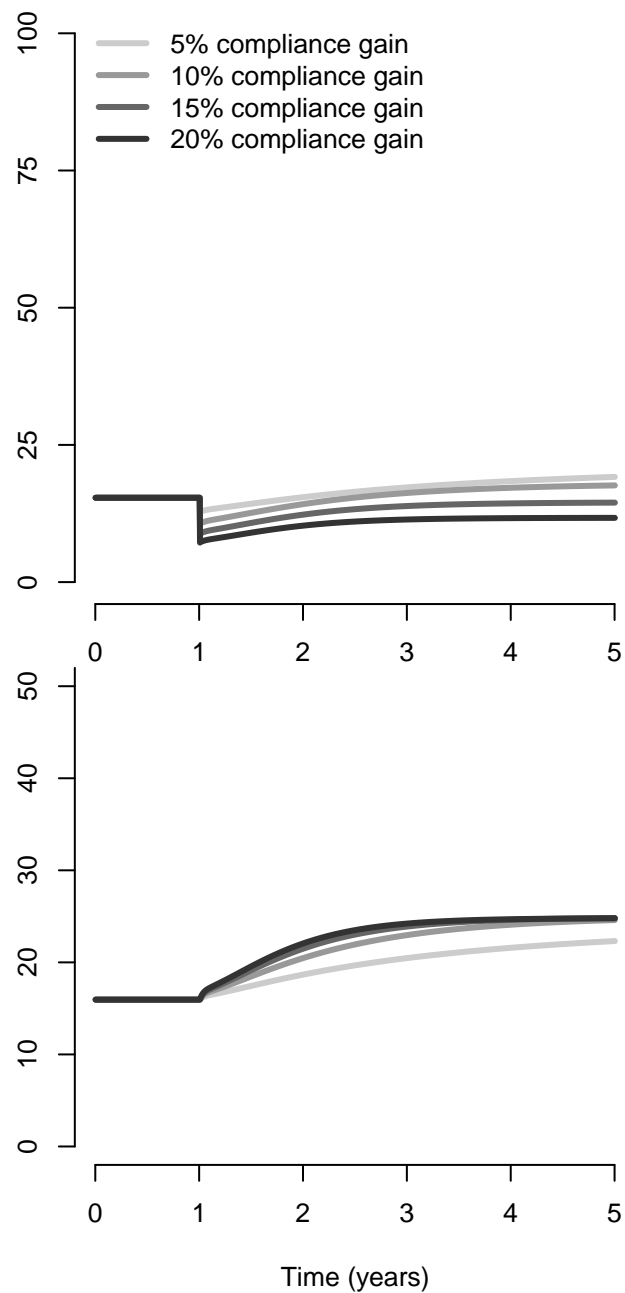

Supplement: Supplementary file 1 [file wellcomeopenres-2-14336-s0000.tgz › b4fdf25b-a701-48c5-81f9-b69b71e19f10.pdf]

## Resistant strain

Adapted to hospital

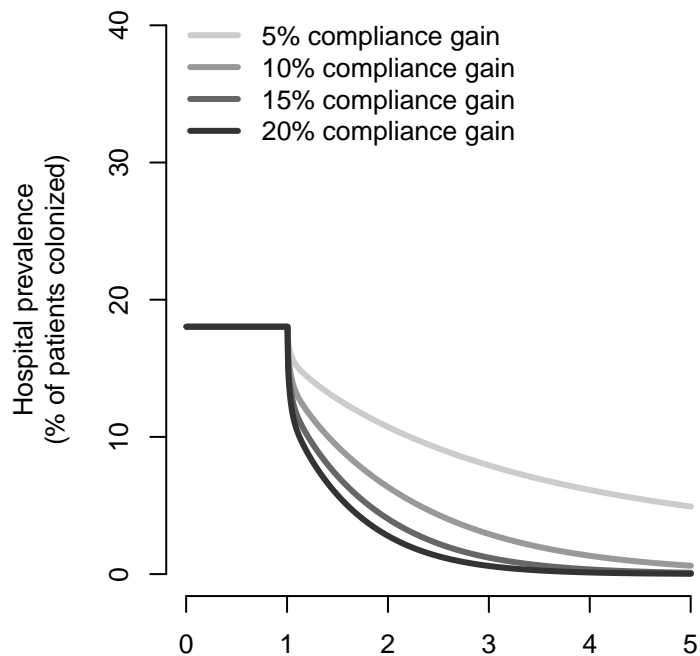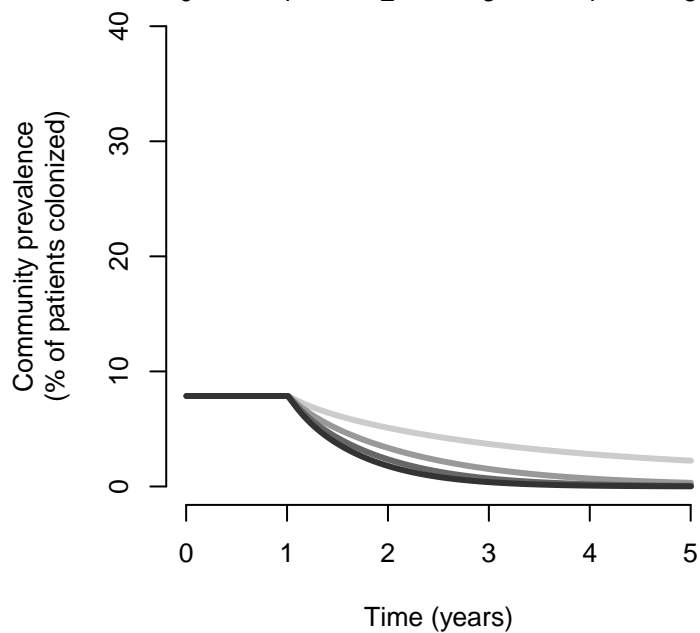

## Sensitive strain

Adapted to community

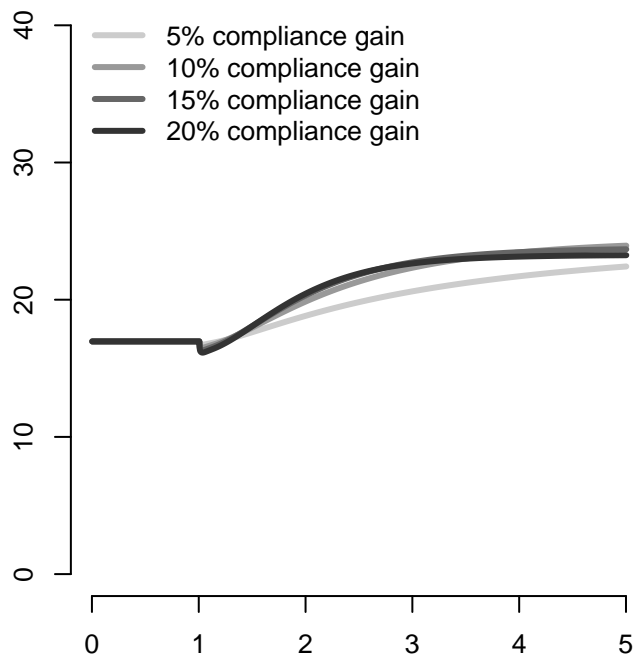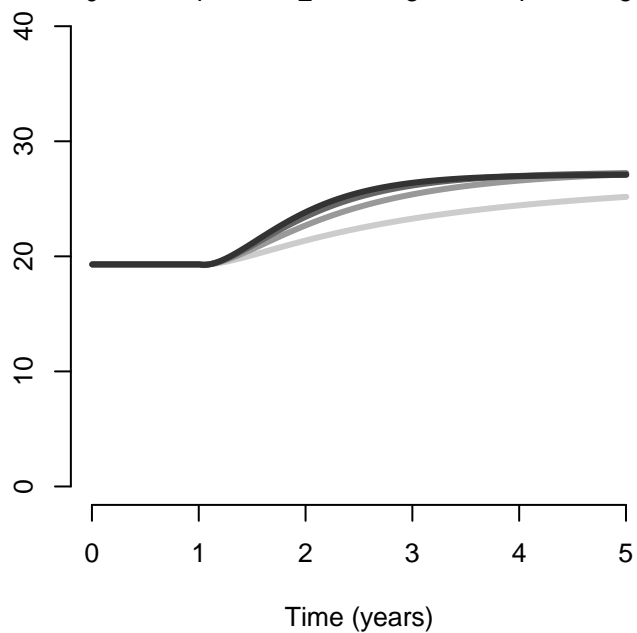

Supplement: Supplementary file 2 [file wellcomeopenres-2-14336-s0001.tgz › 5d6d5454-e550-4791-b030-650551047133.pdf]

Resistant strain  
Adapted to hospital

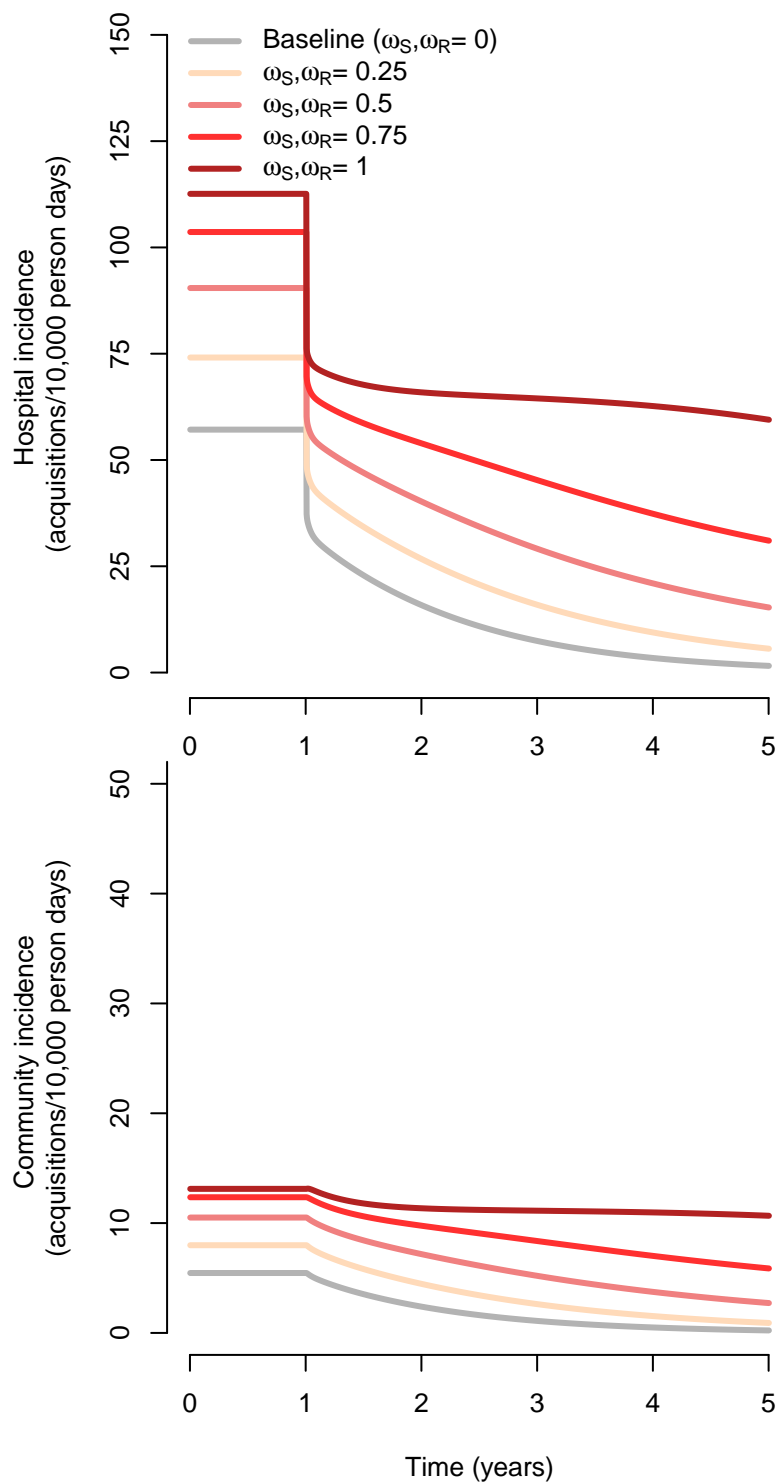

Sensitive strain  
Adapted to community

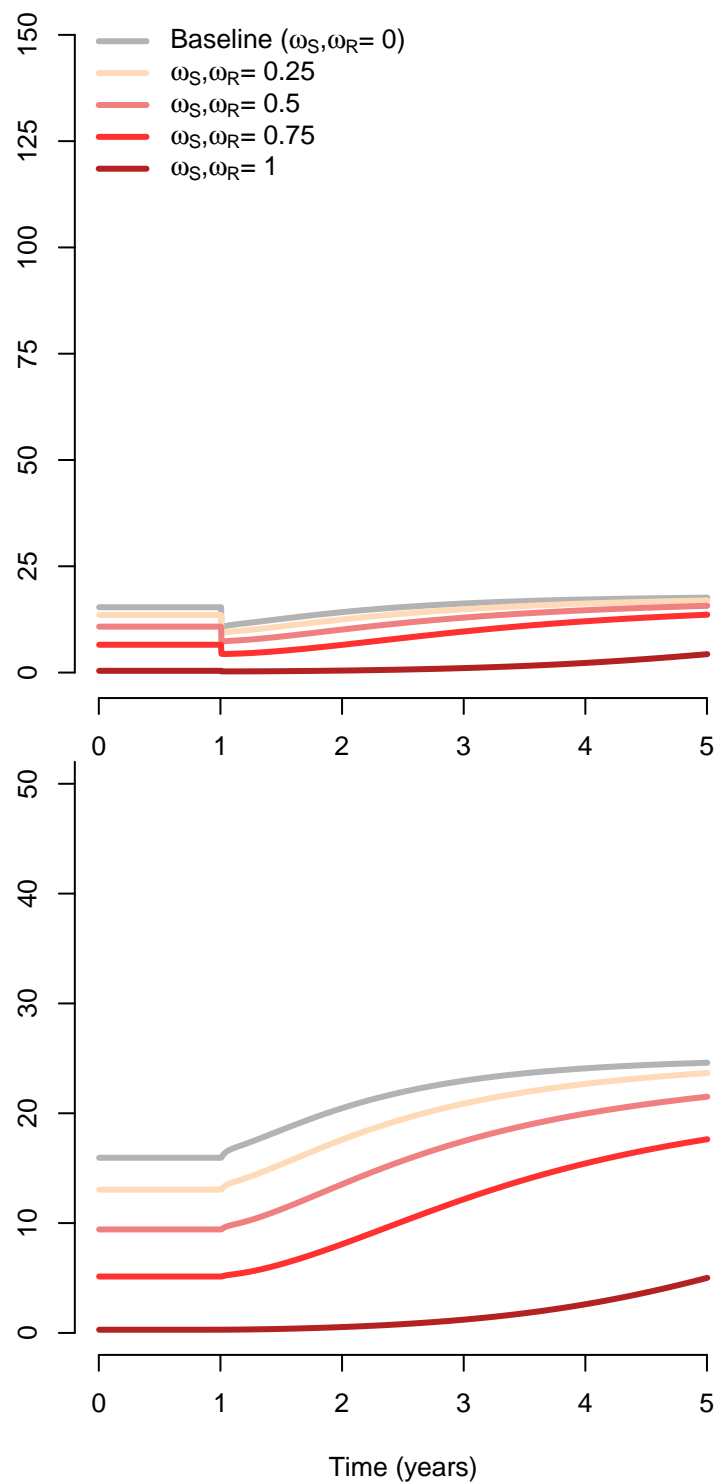

Supplement: Supplementary file 3 [file wellcomeopenres-2-14336-s0002.tgz › fedd1990-b03c-41b0-ba23-aa02ec84f3d5.pdf]

Resistant strain  
Adapted to hospital

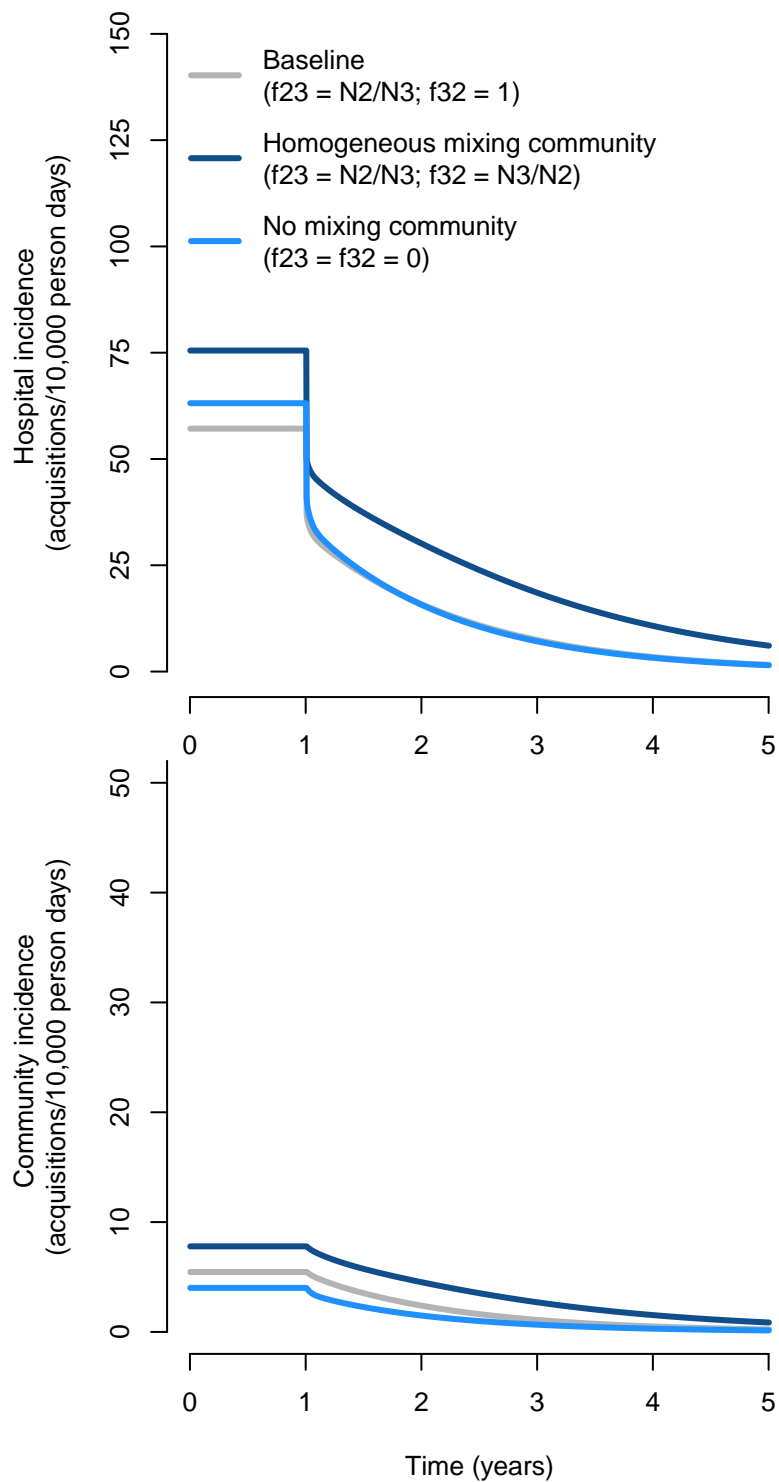

Sensitive strain  
Adapted to community

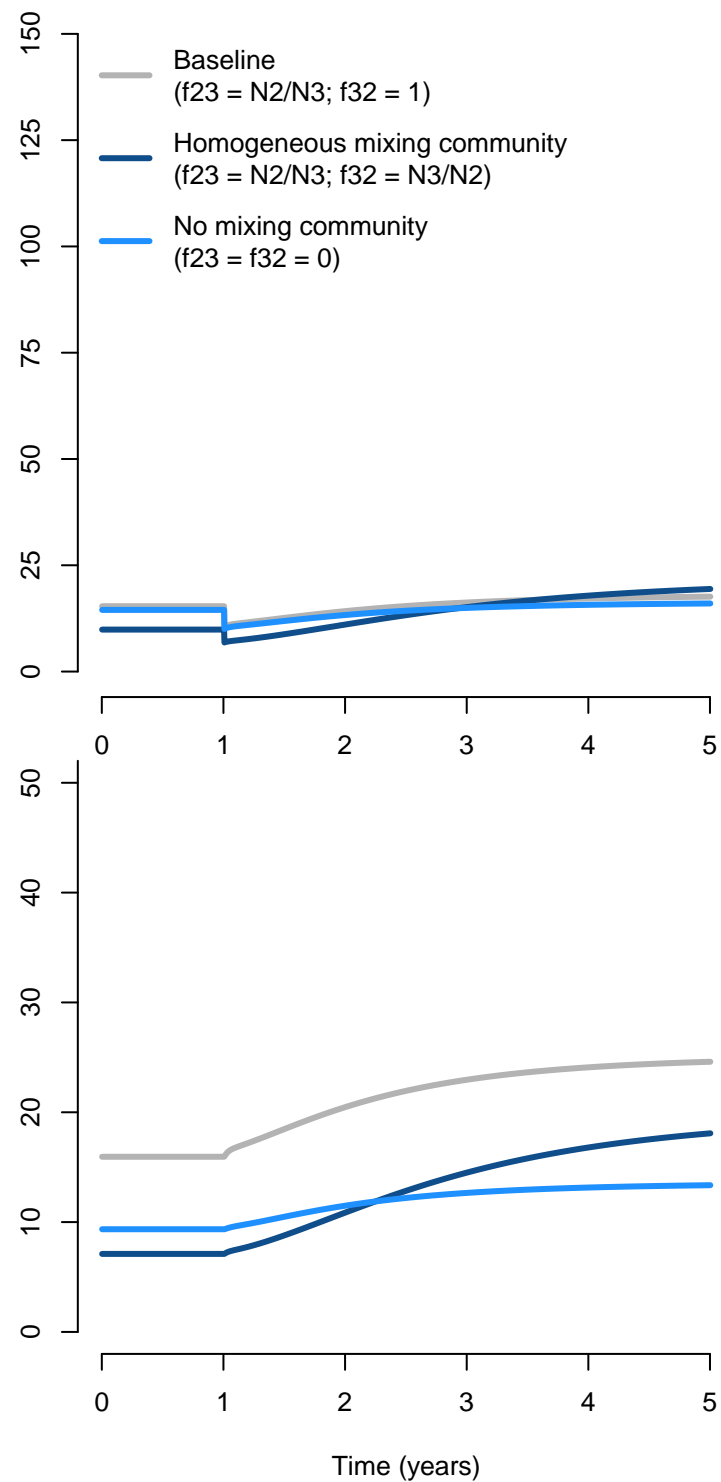

Supplement: Supplementary file 4 [file wellcomeopenres-2-14336-s0003.tgz › c3e04674-7ecb-44ed-a1ad-56fb9e16fee1.pdf]
