# Supplementary material for: Why sensitive bacteria are resistant to hospital infection control
Source: Wellcome Open Res. 2017 Nov 22;2:16. Originally published 2017 Mar 10. [Version 2] doi: 10.12688/wellcomeopenres.11033.2 (PMC5721567; doi:10.12688/wellcomeopenres.11033.2)
Supplement: Peer review history

# Peer review: Referee response for version 2

**DOI:** 10.21956/wellcomeopenres.14336.r28262

**Published:** 2017-12-07

**Authors:** Lulla Opatowski

**Affiliations:** 1 Biostatistics, Biomathematics, Pharmacoepidemiology and Infectious Diseases Unit (B2PHI), UMR1181, Université Versailles Saint Quentin, Institut Pasteur, Inserm, University of Paris-Saclay, Paris, France

The authors have carefully addressed my comments and provided more details on the modelling aspects. I do not have any further comment to make.

 Just a minor typo in the amendment box: “interference” instead of “inference”.

I have read this submission. I believe that I have an appropriate level of expertise to confirm that it is of an acceptable scientific standard.

# Peer review: Referee response for version 1

**DOI:** 10.21956/wellcomeopenres.11901.r21144

**Published:** 2017-06-20

**Authors:** Chris Robertson

**Affiliations:** 1 Department of Mathematics and Statistics, University of Strathclyde, Glasgow, G1 1XH, UK

This paper presents a very interesting transmission dynamic model of resistant and susceptible pathogens in both healthcare and community settings.  It is constructed in such a way as to be able to explore potential reasons why antimicrobial resistant organisms have been declining recently while sensitive versions of the same organism have been increasing or, remaining constant. This elegant model has different rates of transmission depending on the setting and this is the aspect which leads to an explanation of the observed data on reducing rates of MRSA alongside increasing rates of MSSA. 

 I think that the key aspect of the model is that resistant strains are assumed to be better adapted to the hospital setting and so would be expected to generate more secondary cases in hospital than a non resistant strain.  The opposite is assumed to happen in the community.  When an intervention is targeted at reducing transmission in hospitals then this will interfere more with the transmission of resistant organisms in hospital and will have no impact on the transmission in the community.  This crucial assumption is not really justified, nor does it need to be, as the model only seeks to provide a mechanism whereby the observed results can be explained.

 The model equations are standard for this type of model.  This model, like many others, relies on assigning values to a number of parameters.  There are not justified other than to have an R _0_ of 1.5 for resistant strains and 1.4 for susceptible strains.  These are reasonable values and, as this model is an exercise to see if a model can explain the observed results, getting justified parameter estimates for one organism is not really required.

 In some respects the model is similar to the some of the models in 

 Lipsitch *et al.* 2009 ^1^ though co existence of susceptible and resistant strains are not permitted in this model.

 The authors do not claim that this is a model for a disease however I was a little surprising that the resistant strain is eliminated when hand hygiene compliance reaches 55% while coexistence was observed when compliance was 50%. 

 Minor points:

 The model assumes each hospital is associated with a community of 110,000 – this is OK for Scotland with 42 acute hospitals and a population of 5.2 million.  The average size of each hospital is just under 300 beds.  What would be the impact of smaller hospitals and smaller numbers of health care workers per hospital?

 Is the ratio of 100:1000 for health care workers to patients per hospital realistic?

 1 in 10 carriage episodes results in an infection – justification This is the same in hospital and community. However you might expect that immune compromised individuals in hospital who carry a strain might be more likely to develop an infection.

I have read this submission. I believe that I have an appropriate level of expertise to confirm that it is of an acceptable scientific standard.

## References

[1] LipsitchMColijnCCohenTHanageWPFraserC: No coexistence for free: neutral null models for multistrain pathogens. *Epidemics*.2009;1(1) : 10.1016/j.epidem.2008.07.001 2-13 10.1016/j.epidem.2008.07.001 21352747PMC3099423

# Response

**Published:** 2017-11-12

**Authors:** Esther van Kleef

**Affiliations:** Mahidol Oxford Tropical Medicine Research Unit, Thailand

This paper presents a very interesting transmission dynamic model of resistant and susceptible pathogens in both healthcare and community settings.  It is constructed in such a way as to be able to explore potential reasons why antimicrobial resistant organisms have been declining recently while sensitive versions of the same organism have been increasing or, remaining constant. This elegant model has different rates of transmission depending on the setting and this is the aspect which leads to an explanation of the observed data on reducing rates of MRSA alongside increasing rates of MSSA. 

I think that the key aspect of the model is that resistant strains are assumed to be better adapted to the hospital setting and so would be expected to generate more secondary cases in hospital than a non resistant strain.  The opposite is assumed to happen in the community.  When an intervention is targeted at reducing transmission in hospitals then this will interfere more with the transmission of resistant organisms in hospital and will have no impact on the transmission in the community.  This crucial assumption is not really justified, nor does it need to be, as the model only seeks to provide a mechanism whereby the observed results can be explained.

The model equations are standard for this type of model.  This model, like many others, relies on assigning values to a number of parameters.  There are not justified other than to have an R _0_ of 1.5 for resistant strains and 1.4 for susceptible strains.  These are reasonable values and, as this model is an exercise to see if a model can explain the observed results, getting justified parameter estimates for one organism is not really required.

In some respects the model is similar to the some of the models in 

Lipsitch *et al.* 2009 ^1^ though co existence of susceptible and resistant strains are not permitted in this model.

1.The authors do not claim that this is a model for a disease however I was a little surprising that the resistant strain is eliminated when hand hygiene compliance reaches 55% while coexistence was observed when compliance was 50%. 

**This is over the 5-year period modelled, as presented in Figure S1 and Figure S2. The lower the improvement in hand hygiene, the more gradual the decline in resistant bacteria. Running the model over a 10-year period shows that eventually elimination of the resistant strain will be reached under 50% hand hygiene compliance as well. However, we considered a 5-year time horizon sufficient to illustrate the underlying dynamics. We have added the following text to the result section:**

*“…increasing it further to 55% induced a more rapid decline in both the hospital and community reservoir and successfully eliminated the resistant strain* ***within the five year time period simulated*.”**

Minor points:

2.The model assumes each hospital is associated with a community of 110,000 – this is OK for Scotland with 42 acute hospitals and a population of 5.2 million.  The average size of each hospital is just under 300 beds.  What would be the impact of smaller hospitals and smaller numbers of health care workers per hospital?

**With smaller population sizes (and smaller hospitals), stochastic effects would become increasingly important and the relative magnitude of fluctuations in each hospital would increase (in proportion to the reciprocal of the square root of the population size).  Such stochastic effects are not accounted for in our paper as the key intention was to shed light on how hospital interventions could lead to long-term trends in large populations. Stochastic models would lead to the same broad conclusions, but in any given simulation the trends might be obscured by stochastic fluctuations (particularly when populations are small).  We have added the following sentence listed in bold to the discussion:**

*Our model also ignores a lot of host and pathogen heterogeneity **, and we did not account for stochastic effects. In small populations of single hospitals, chance events are likely to play an important role in the transmission dynamics of pathogens. **However, we can think of no plausible mechanism by which incorporation of more biological realism would in any way alter our primary conclusion. Though our framework allows for further complexity, the purpose here was to demonstrate that the divergent effects of infection control interventions on resistant and sensitive models could be explained even with a simple model.*

3. Is the ratio of 100:1000 for health care workers to patients per hospital realistic?

**The EU FP7 framework RN4cast study surveyed practicing nurses in nine European countries in 2012, and hence provided insight in acute Trust nursing staff ratios in these countries, including England**[1,2] **. For England, ~3000 nurses from 31 Trusts, 46 hospitals participated in the study. Patient to nursing staff ratios were highly variable across the sampled Trusts in England, with an overall average of per 1 one patient, 8.8 [Range: 5.5 – 11.5] registered nurses **[2] **. Moreover, levels of 1:8 were found during day time and 1:11 during night time on average**[1] **. Across all participating countries, the patient-to-nurse ratio was 1:8.3 [2.4 – 17.9] **[2] **. We are aware that these figures include nurses only, while not accounting for other healthcare workers. As nurses account for the largest fraction of caring staff, we believe that our patient healthcare worker ratio of 1:10 is not unrealistic. However, we wish to emphasise that lower patient-to-healthcare worker ratios would not change our findings.**

**We have added the above listed references to Table 2.**

4. 1 in 10 carriage episodes results in an infection – justification. This is the same in hospital and community. However you might expect that immune compromised individuals in hospital who carry a strain might be more likely to develop an infection.

**Fundamentally, we have presented a model of carriage dynamics and the assumption is that clinical infections increase in line with the number of carriers.  The key results presented do not depend on the proportion of colonized patients who develop an infection. For example, in figure 3, incidence refers to the "** **incidence of new acquisitions (symptomatic and asymptomatic)** **" and figure 4 also refers to changes in incidence of acquisitions of both symptomatic and asymptomatic infection (apologies if this was not clear). The exception is figure 2 where we are attempting to simulate something like the incidence data we observe (i.e. samples submitted to the Oxford University Hospitals NHS Trust that tested positive for *C. difficile. *This hospital does all the *C. difficile *testing in Oxfordshire, including samples submitted by GPs, community hospitals and other providers**[3] **), though in this case we report only changes in these incidence rates associated with an intervention (i.e. incidence rate ratios) rather than absolute numbers to aid comparison with reference 4.  The net result is the expected values of these IRRs will be insensitive to the risk of clinical infection given carriage in hospital and community, but the absolute numbers (and therefore degree of dispersion) will be sensitive to this. We agree that difference in case mix between hospitalised individuals and individuals residing in the community might lead to a difference in likelihood of developing infection. In addition, hospital-acquired *C. difficile *cases are probably more likely to be reported than community-acquired cases.  Indeed, this may explain why the simulations are giving wider dispersion for the IRR for hospital linked cases than observed data but somewhat narrower dispersion for the community linked cases (for which we may be overestimating the proportion seen in hospital). To address this issue** **we allowed for different constants of proportionality for hospital and community-linked infections, and, by default, assumed that hospital-acquired cases were 5 times more likely to be reported as community-acquired cases. Figure 2 has been updated accordingly.**

**In addition, the method section now reads:**

*“We assumed the number of new infections reported with a hospital link or a community was proportional to the cumulative number of acquisitions in the hospital **or **community in each of the two time periods”*

**In explaining our calculated observed infections we now say:**

***“…and assuming 1 in 10 carriage episodes acquired in hospital resulted in a reported infection. This was 1 in 50 for community-acquired episodes. Hence we allowed for differences in reporting rates in both settings as well as heterogeneity in case-mix affecting the likelihood of developing an infection.”***

**Moreover, we have changed the caption of Figure 4 in line with figure 3, the caption now reads:**

***“Annual incidence rate ratios of new acquisitions (symptomatic and asymptomatic) associated with an infection control intervention under different levels of adaptation of sensitive and resistant strains to hospital and community settings”***

References:

1. Ball J, Pike G, Griffiths P, Rafferty A, Murrells T. RN4CAST Nurse Survey in England. London; 2012.

2. Aiken L, Sloane D, Bruyneel L, Van Den Heede K, Griffi P, Busse R, et al. Nurse staffing and education and hospital mortality in nine European countries: a retrospective observational study. Lancet. 2014;383:1824–30.

3. Dingle K, Didelot X, Eyre D, Stoesser N, Golubchik T, Harding R, et al. Effects of control interventions on Clostridium difficile infection in England: an observational study. Lancet Infect Dis. 2017;

# Peer review: Referee response for version 1

**DOI:** 10.21956/wellcomeopenres.11901.r21315

**Published:** 2017-04-27

**Authors:** Lulla Opatowski

**Affiliations:** 1 Biostatistics, Biomathematics, Pharmacoepidemiology and Infectious Diseases Unit (B2PHI), UMR1181, Université Versailles Saint Quentin, Institut Pasteur, Inserm, University of Paris-Saclay, Paris, France

This article aims at investigating the impact of implementing unspecific control measures, such as hand hygiene, on the spread of antibiotic resistant and antibiotic sensitive bacteria in hospitals, using mathematical modelling. In this theoretical study, a new deterministic model based on ODEs is numerically simulated under different hand hygiene scenarios. For each scenario, the resulting annual incidence ratio is calculated for hospital- and community-acquired infections with resistant and sensitive bacteria. The simulation results suggest that, counter-intuitively but in accordance to the observations from recent years, infection control interventions such as hand hygiene can have discordant effects on resistant and sensitive strains, even if they do not target specifically one or the other.

 This is a very clear and well written article and I really enjoyed reading it. The question addressed is of high importance in a context where antimicrobial resistance keeps increasing and limited number of drugs and interventions are available to control it. Understanding better the respective impact of control measures is therefore essential to optimize their implementations and also interpret the observed trends. However, to make the presented results more convincing and interpretable, some clarification about the model is needed, in addition to sensitivity analysis on the model parameters.

Main comments

1. Modelling health care workers (HCW) colonisation. An originality of the model is that it specifically formalizes the patient-HCW transmission. Here, HCWs are classified either as non-hand-carriers or as hand-carriers. In the model, hand hygiene is therefore assumed to directly impact to directly clear HCW carriage in compliant individuals (eg at a proportion of 50%). Several epidemiological studies have shown that, in the case of S. aureus at least, proper (nasal) colonization is frequent in HCW. I would expect that, for those HCW properly carrying the bacteria, efficient hand hygiene may impede transmission to others by clearing hand carriage, but would not clear colonisation. They would not need to be recolonized through contact with patients to become again S or R carriers the next day. On the contrary, for purely transient hand carriers HCWs, I would expect that hand hygiene completely removes the bacteria from the hand and entire body. In that case, new acquisition from patient would be necessary for them to become carrier again. Can the authors comment on that point? In particular, how is HCW’s duration of carriage handled in the model?
2. Parameters table. The table needs some clarifications and references. (1) I did not understand the values of the following rates: tau, gammaR, and gammaS: is the rate or the duration depicted in the last column? It looks more like the reciprocal duration, despite the unit is given in day-1. (2) I think a % is lacking in the last raw of the table. (3) Can you please explain the calculation of f23, this is not clear to me. (4) If I understand well, carriage is assumed to last for 400 days. This is quite long and may have consequences on the resulting trends obtained in the simulations. Can the authors provide a justification for this value and carry out some sensitivity analysis on this parameter? (5) Can you provide some justification about the values of p set to 10?
3.  Bacterial interference. This is not clear whether the authors finally assumed some competition for colonisation between the strains or not. On the schematic representation of the model, no “superinfection” is assumed, but this mechanism is described in the Methods section. If w=0 as indicated in Table2, then full competition is assumed between the strains. This hypothesis is strong and may have some influence on the resulting trends. My intuition is that this strong assumption may provide more chance to S strains to spread in hospitals when R strains are removed by intervention. Could the authors carry out some sensitivity analysis on the impact of that parameter?
4. Transmission rate. Could you provide more details about beta calculation for the different strains in the different settings according to R0? Also, in the section “Importance of the degree of strain adaptation…”, “when increasing the transmission that occurs in hospital”, could you provide the corresponding values for beta?
  Similarly, when investigating the importance of environmental adaptation, how did you process to vary “the level of transmission in both settings for each of the two strains, while keeping the overall basic reproduction number for R and S strains constant…”?
5. Model equations. Frequency dependent hypothesis is assumed in the ward which looks realistic. However, in some equations, this rule does not apply; it would require some explanation. In equations describing the hcwS and hcwR derivatives, the denominator of the transmission term is for example Nhcw.
6. Community transmission. The expressions of the force of infections for patients are not totally clear to me either. In particular, I don’t understand the term beta_R3xR3xf23/N2. Given the definition of f23, this expression is actually equivalent to beta_R3xR3/N3, which makes more sense to me. In general, it would be good if more details were provided to explain the model community transmission. It was not clear to me what the authors meant by “the model allowed for the possibility of assortative mixing within population 2 and 3”. Could you provide a mixing matrix to make clear the transmission between the 3 (or 4) populations? Similarly, expressions of lambda_R3, lambda_S1, lambda_S2 and lambda_S3 would need some more explanation. Why is it divided respectively by N3, N2 and N3?
7. Annual incidence rate ratio. Could you provide an equation for the calculation of IRR as a function of the measured outputs from the results?
8. Interpretation of results about the dynamic after hospital infection control. To make the interpretation of Figure 3 more convincing, it would be important to disentangle what processes come from the community- to-community transmission, the hospital- to-community (ie community importations) transmission, the hospital- to-hospital transmission and the community-to-hospital (ie hospital importations) transmission. Could the authors present, in addition to Figure3, the incident cases coming from these different processes?
9. The modelled hospital population is 1000 patients and 100 HCWs. The proposed model is deterministic. How would stochasticity impact the results?

 

Minor comments

1. Does R0 define the number of secondary cases of infection or colonisation? As transmission occurs through colonization my choice would go for that one but in the main text and Table 2 legend, the authors mention “infection”. In addition, as R0 is actually defined in a setting with already 40% hand hygiene at baseline, this is actually not the strict basic reproductive number of the bacteria. I therefore suggest naming it reproductive number (R) which seems more correct to me.
2. Table1. hCWR and hCWS notations do not match with notations in the model depicted in fig1. Could the authors check they use the same notations?

I have read this submission. I believe that I have an appropriate level of expertise to confirm that it is of an acceptable scientific standard, however I have significant reservations, as outlined above.

# Response

**Published:** 2017-11-12

**Authors:** Esther van Kleef

**Affiliations:** Mahidol Oxford Tropical Medicine Research Unit, Thailand

This article aims at investigating the impact of implementing unspecific control measures, such as hand hygiene, on the spread of antibiotic resistant and antibiotic sensitive bacteria in hospitals, using mathematical modelling. In this theoretical study, a new deterministic model based on ODEs is numerically simulated under different hand hygiene scenarios. For each scenario, the resulting annual incidence ratio is calculated for hospital- and community-acquired infections with resistant and sensitive bacteria. The simulation results suggest that, counter-intuitively but in accordance to the observations from recent years, infection control interventions such as hand hygiene can have discordant effects on resistant and sensitive strains, even if they do not target specifically one or the other.

This is a very clear and well written article and I really enjoyed reading it. The question addressed is of high importance in a context where antimicrobial resistance keeps increasing and limited number of drugs and interventions are available to control it. Understanding better the respective impact of control measures is therefore essential to optimize their implementations and also interpret the observed trends. However, to make the presented results more convincing and interpretable, some clarification about the model is needed, in addition to sensitivity analysis on the model parameters.

Main comments

1. Modelling health care workers (HCW) colonisation. An originality of the model is that it specifically formalizes the patient-HCW transmission. Here, HCWs are classified either as non-hand-carriers or as hand-carriers. In the model, hand hygiene is therefore assumed to directly impact to directly clear HCW carriage in compliant individuals (eg at a proportion of 50%). Several epidemiological studies have shown that, in the case of S. aureus at least, proper (nasal) colonization is frequent in HCW. I would expect that, for those HCW properly carrying the bacteria, efficient hand hygiene may impede transmission to others by clearing hand carriage, but would not clear colonisation. They would not need to be recolonized through contact with patients to become again S or R carriers the next day. On the contrary, for purely transient hand carriers HCWs, I would expect that hand hygiene completely removes the bacteria from the hand and entire body. In that case, new acquisition from patient would be necessary for them to become carrier again. Can the authors comment on that point? In particular, how is HCW’s duration of carriage handled in the model? 
**We recognise that both types of carriage have been found among healthcare workers, at least in the case of MRSA. A review of the literature **[4] **revealed a wide array of studies with some finding persistent carriage to be more common, whereas other studies concluded transient carriage is the most frequent type of carriage among healthcare workers. The findings of these studies are likely to be setting dependent and, as concluded by Albrich and Harbarth **[4] **, sensitive to misclassification bias (e.g. incorrectly defining transient carriage as persistent carriage), and are, in any case, not informative about the significance of longer-term staff carriage for hospital transmission dynamics. In this model, we assumed that persistent carriage by healthcare workers does not play a significant role in hospital transmission and can be neglected. Clearly, as with other model assumptions, this is a simplification, and we acknowledge that there are case reports of outbreaks with a plausible link to long-term staff carriers. However, our assumption is supported by a prospective carriage study of MRSA in adult and paediatric ICUs using whole genome sequencing to determine possible transmission pathways **[5] **. This study showed frequent patient acquisition events of MRSA with closely related strains shared between overlapping patients, strongly suggesting patient-to-patient transmission. In contrast, while MRSA was recovered from nasal swabs from four healthcare workers, there was only a single patient MRSA acquisition that could have been plausibly related to known staff carriage (based on the whole genome sequencing data). Since patients were not mobile, patient-to-patient transmission events are likely to represent either hand-borne or air-borne transmission. Studies from the 1960s suggest that the latter plays a relatively minor role in *S. aureus* transmission in hospital settings (see, for example, **[6,7] **). Since the purpose of our model is to illustrate the potential differential effects of infection control measures on sensitive and resistant strains (and not to make strong claims for potential impact of different control measures), for simplicity we chose to focus on what appears to be the dominant mode of transmission (at least for *S. aureus*, other organisms are less well studied) rather than adding non-essential complexity by accounting for other transmission pathways.  Therefore, following hand hygiene, healthcare worker carriage is cleared and new contact with contaminated patients is required for healthcare workers to become carriers again. Persistent colonization such as the nasal colonization the reviewer is referring to is not included in this model. Hence no explicit colonization time for healthcare workers is assumed. We have now added the following sentence to our methods:**
***“Persistent carriage of bacteria such as MRSA has been reported among healthcare workers, though is commonly found to be transient”.*** **Followed by the text already present: “ *Therefore, healthcare workers in turn were assumed to become transiently contaminated through patient contact. Hand hygiene performed by a contaminated healthcare worker was assumed to clear this contamination”***
**In addition, we have added the following text to our discussion:**
***Moreover, for simplicity we chose to focus on what appears to be the dominant mode of transmission (at least for S. aureus, other organisms are less well studied). Since hospitalised patients are generally not mobile, patient-to-patient transmission events represent either hand-borne or air-borne transmission. Studies from the 1960s suggest that the latter plays a relatively minor role in S. aureus transmission in hospital settings [24,25].”***
2. Parameters table. The table needs some clarifications and references. (1) I did not understand the values of the following rates: tau, gammaR, and gammaS: is the rate or the duration depicted in the last column? It looks more like the reciprocal duration, despite the unit is given in day-1. (2) I think a % is lacking in the last raw of the table. (3) Can you please explain the calculation of f23, this is not clear to me. (4) If I understand well, carriage is assumed to last for 400 days. This is quite long and may have consequences on the resulting trends obtained in the simulations. Can the authors provide a justification for this value and carry out some sensitivity analysis on this parameter? (5) Can you provide some justification about the values of p set to 10
   
  1. **We thank the author for spotting this error, the last column should indeed depict the rates for tau, gammaR and gammaS. We have now changed the values to reflect the reciprocal duration. **
  2. **The % sign is now added to the last row of table 2  **
  3. **See comment 6**
  4. **We belief 400 days of carriage is a plausible value to represent the dynamics of MRSA. For example, Scanvic et al, CID 2001 found a median duration of carriage of 8.5 months among patients readmitted to hospital, following an exponential distribution**[8] **. This would result in an average duration of:** y _in_ = (8.5/12)*365*1/log(2) = 373 **days.**
**For other major pathogens this is less well known, but, in the case of for example *C. difficile, *likely to be shorter. Our key results however, are not affected by this; in Van Kleef et al LID 2017**[9] **we show an example where we assumed a shorter duration of carriage (200 days) and were able to produce similar findings and conclusions. We feel that presenting additional sensitivity analysis here would make the paper harder to read and potentially obscure the key message.**
**However,** **we have added a reference to our analysis using different parameter values, including a shorter duration of carriage **[9] **to the discussion:**
***“For example, assumptions about carriage duration, mixing of community populations, and the degree of bacterial interference between the two strains can easily be altered (and will not change our main conclusions, as shown in respectively [20], Figure S3, Figure S4)”*** **Furthermore,** **we added the reference of Scanvic et al to table 2.**
  5. **The rational behind the different transmission probabilities of patients and healthcare workers relates to our assumption explained under comment 1. We assume that healthcare workers were transiently hand-carriers of bacterial pathogens, whereas patients were fully colonised. For patients, this could be e.g. on the skin, wounds or nasal carriage. We translated these different degrees of carriage in different risks of transmission.** **We fully agree with the reviewer that a justified value for *p *based on scientific evidence would be important if we were interested in quantifying effects of hand hygiene interventions specifically. However, we are just using hand hygiene as a specific example of an intervention that interrupts transmission in the hospital but not the community for resistant and sensitive strains equally.** **To make the latter clearer** **, we h** **ave added the following in bold to our introduction:**
*“We explicitly model a hospital hand hygiene intervention **as an example of a non-specific infection control measure **and evaluate the impact of this intervention on the incidence of hospital and community acquisitions of antibiotic-resistant and antibiotic-sensitive strains.”*
3. Bacterial interference. This is not clear whether the authors finally assumed some competition for colonisation between the strains or not. On the schematic representation of the model, no “superinfection” is assumed, but this mechanism is described in the Methods section. If w=0 as indicated in Table2, then full competition is assumed between the strains. This hypothesis is strong and may have some influence on the resulting trends. My intuition is that this strong assumption may provide more chance to S strains to spread in hospitals when R strains are removed by intervention. Could the authors carry out some sensitivity analysis on the impact of that parameter?
**e apologies that we did not make our assumptions regarding bacterial inference clear in our manuscript. We do assume competition for colonization between strains. This is specified by the parameters w _s_** **and w _r_** **(see table 2) which by default were set to 0 (implying carriage of one strain completely blocks acquisition of another i.e. complete bacterial interference). In response to this comment we have now conducted an additional sensitivity analysis where we assume w _S_,w _R_ = 0.25, 0.5, 0.75 and 1 respectively. The results are presented in Figure S3.**
**We find that at lower levels of bacterial interference (i.e. higher** ***ω _s_*** **and** ***ω _R_*** **), the incidence of the resistant strain in both the hospital and the community is higher compared to baseline, whereas the incidence of the sensitive strain is lower in both settings (Figure S3). This relates to the higher R _n _of the resistant strain, giving it a competitive advantage over the sensitive strain, which is further enhanced when replacement infection is allowed for. At w _S_,w _R_ = 1, this means the sensitive strain does not coexist with the resistant at baseline hand hygiene levels of 40%. However, after implementation of the hand hygiene intervention, the sensitive strain will emerge even under these conditions. Thus, what is shown is that regardless of the level of bacterial interference, the overall conclusions remain unchanged: hospital infection control can have discordant effects on resistant and sensitive bacteria provided heterogeneity is present in their respective environmental adaptations.**
**Just to clarify, while we do consider competition we do not consider superinfection (i.e. co-infection with both sensitive and resistant strains**[10] **) and we state this in the methods section “ *Individuals could not be co-infected with resistant and sensitive strains”. *Including this would greatly add to the complexity of the model, reduce readability and, we feel, obscure the key messages.**
**We have added the following text in bold to the discussion section:**
*“For example, assumptions about carriage duration and the degree of bacterial interference between the two strains can easily be altered **(and will not change our main conclusions, Lancet ID paper, Figure S3, Figure S4)”***
**Moreover, we have added the term ‘replacement infection’ to our methods, which now reads:**
***“we allowed for bacterial interference between the two strains so that colonization with one strain reduced the risk of acquisition of the other strain, i.e. replacement infection*** **”.**
4. Transmission rate. Could you provide more details about beta calculation for the different strains in the different settings according to R0? Also, in the section “Importance of the degree of strain adaptation…”, “when increasing the transmission that occurs in hospital”, could you provide the corresponding values for beta?Similarly, when investigating the importance of environmental adaptation, how did you process to vary “the level of transmission in both settings for each of the two strains, while keeping the overall basic reproduction number for R and S strains constant…”?
**We varied the values of beta_R _n_ (where n = population 1, population 2, population 3)** **to give values of R0 = 1.5 an beta_R _s _to give values of R0 = 1.4 keeping all other parameter values constant and ran the model at equilibrium. If at model equilibrium, the strain adaptation to each of the respective environments was at the desirable level, these values were chosen for model parameterisation. Here, the degree of strain adaptation was defined as the fraction of new acquisitions of strain i occurring in setting n (hospital or community) respectively on each day t.**
**The corresponding values for all beta_i _n _used to produce figure 4 are available at https://github.com/esthervankleef/Two_strain_model_published. We have added the following text in bold to the method section:**
*“Increasing adaptation of the resistant strain to the hospital environment (i.e. increasing the proportion of resistant transmission that occurs in hospital **by changing the values of the transmission parameters (beta_i _n_) **while keeping the basic reproduction number **and all other parameters constant),***
**Also, we added the following text to the legend of Figure 4.**
***“For corresponding transmission parameter values see https://github.com/esthervankleef/Two_strain_model_published”***
5. Model equations. Frequency dependent hypothesis is assumed in the ward which looks realistic. However, in some equations, this rule does not apply; it would require some explanation. In equations describing the hcwS and hcwR derivatives, the denominator of the transmission term is for example Nhcw.
**The reason why the denominator for hcw _S_ and hcw _R_ derivatives is N _hcw_ follows from the structure of our model, which assumes healthcare workers are the pathogen vectors.  Similar to Cooper et al (1999)**[11] **we assume that in hospital, all transmission events between patients is caused by a contact from a transiently colonized HCW. Similarly, HCW acquire transient hand-contamination only by touching colonized patients.**
In our model, beta_R _1 _= the probability that an uncolonized patient becomes colonized on contact with a colonized healthcare worker, whereas p*beta_R _1 _= the probability that an uncolonized healthcare worker becomes colonized with a resistant strain on contact with a colonized patient (where rho = ratio of probability of patient-to-HCW transmission vs HCW-to-patient transmission).
Hence, the rates at which contacts are made which can potentially result in colonization with a resistant strain is beta_R _1_*U _1_ for patients (i.e. a function of beta_R _1 _and the number of uncolonized patients U _1_), and p*beta_R _1_*R _1 _for healthcare workers (i.e. a function of p *, *beta_R _1 _and **the number of colonized patients R _1_). Only a fraction of these contacts will result in transmission. For patients this is the fraction of contacts which are with colonized healthcare workers (hcw­ _R_/N _hcw_) _._ For healthcare workers, it is those contacts in which the healthcare worker is uncolonized that will result in transmission (N _hcw _– hcw _R – _hcw _s_)/ N _hcw_).**
As a result, the rate of colonization for patients and healthcare workers is beta_R1*U _1_*hcw _R_/N _hcw_ and p*beta_R _1_*R _1_(N _hcw_ – hcw _R_ – hcw _s_)/N _hcw _respectively.
**In the community we do assume direct transmission between individuals, hence here the population size of the susceptible hosts is used as a denominator while following the assumption of frequency dependent transmission of Otto and Day**[12] **, further explained under comment 6.**
**Of note: the parameter p was left out of equations 10 and 11, and has now been added.**
6. **C**ommunity transmission. The expressions of the force of infections for patients are not totally clear to me either. In particular, I don’t understand the term beta_R3xR3xf23/N2. Given the definition of f23, this expression is actually equivalent to beta_R3xR3/N3, which makes more sense to me. In general, it would be good if more details were provided to explain the model community transmission. It was not clear to me what the authors meant by “the model allowed for the possibility of assortative mixing within population 2 and 3”. Could you provide a mixing matrix to make clear the transmission between the 3 (or 4) populations? Similarly, expressions of lambda_R3, lambda_S1, lambda_S2 and lambda_S3 would need some more explanation. Why is it divided respectively by N3, N2 and N3?
**This is a good point. We recognize that our baseline assumptions regarding the mixing of our population need further clarification.** **In our description of the force of infection, we followed the definition of Otto and Day, who decompose the frequency dependent incidence rate, i.e. the rate at which new infections occur, in three components **[12] **:**
**The rate of contact with other individuals in the population ( *c*) which are of an appropriate type for transmission to be possible if one of the hosts is infectious**
**The probability that the contact is indeed with a susceptible host U ( *p, *assumed to be U(t)/N(t))**
**The probability that a contact between an infectious and susceptible host leads to successful transmission ( *v*)**
**In the case of frequency dependent transmission, c is assumed constant, hence the effective contact rate or transmission coefficient beta = cv. Assuming frequency dependent transmission, and the definition of Otto and Day, the mixing matrix for the community population should be as follows, where the beta terms are equivalent to the effective contact rates, and e.g. f _23_/beta_R _3_** **reflects that the effective contact rate between individuals of population 2 and population 3 is a fraction of the effective contact rate between individuals in population 3.**
Equation 1
**Multiplying these by the number of infected individuals in each population and the probability *p _i_*that the contact is indeed with a susceptible host (U _Ri _/N _i_) would result in the following incidence rates:**
Equation 2
**As our lambdas represent the force of infections, i.e. the rate at which a susceptible individual becomes infected, we replaced p _i_** **of the incidence rates** **with the probability that the contact is with one specific susceptible host, i.e.** **and** **, resulting in:**
Equation 3
**The same logic applies to lambda_S _2_** **and** **lambda_S _3._**
**We realise our description of the f _23_ and f _32_ in table 2 was not entirely correct and would be better described as given below. Moreover, the reference of our frequency dependent transmission has been added to the method section, which now reads:**
***“We assumed frequency-dependent transmission [10]. The model allowed for the possibility of assortative mixing within populations 2 and 3, where the effective contact rate of strain i (beta_in) between individuals within a population is a fraction of the effective contact rate between individuals across populations: “***
Equation 4
**We have changed the description of f _23_ and f _32_ in Table 2 to:**
***f23 = The ratio of the effective contact rate in N2 from someone in N3 to the effective contact rate in N3 from someone in N3 (where 1 implies that on contact, someone in N3 is causing new infections in N3 and N2 at the same rate. Of note, as N3 > N2, f23 = N2/N3 assumes the same per capita infection rate, i.e. homogenous mixing.***
***f32 = The ratio of the effective contact rate in N3 from someone in N2 to the successful contact rate in N2 from someone in N2 (where 1 implies that on contact, someone in N2 is causing new infections in N2 and N3 at the same rate. Of note, as N3 > N2, f32 = N3/N2 assumes the same per capita infection rate, i.e. homogenous mixing.***
**With regards to our assortative mixing assumption, as described in the above definitions, by defining f23 = N2/N3 and f32 = 1, we assume that individuals in N3 are equally likely to infect an individual in N2 as they are to infect a given individual in N3, whereas setting f32 to 1 implies a higher per capita rate of infection in N2, given N2 < N3, thus individuals in N2 are more likely to infect an individual in N2 than an individual in N3.** **Baseline values of these parameters were chosen to reflect perceived heterogeneities in contacts (e.g. assuming a scenario of LTCFs (N2) vs the general population (N3) where those in N2 will be exposed preferentially to those in N2, but, considering some in N3 will be carers to N2, N3 will mix equally with N2 and N3. These assumptions are not critical to our results, as shown by** **a sensitivity analysis, where we modelled the following additional scenarios:**
**Fully homogenous mixing (f23 = N2/N3, f32 = N3/N2)**
**Fully assortative mixing (f23= f32=0) **
**The results of this analysis are presented in Figure S4. We have added the following text to the discussion section:**
*“For example, assumptions about carriage duration and the degree of bacterial interference between the two strains can easily be altered **(and will not change our main conclusions, Lancet ID paper, Figure S3, Figure S4)”***
7. Annual incidence rate ratio. Could you provide an equation for the calculation of IRR as a function of the measured outputs from the results?
**The annual incidence ratio represents the ratio of the number of new infection in the year pre-intervention (T _0_) to the number of new cases in the first year post-intervention (T­ _1_). As mentioned in the method section, Y _in _represents the actual number of observed infections of strain i in population n, which is proportional to the cumulative number of acquisitions in each population in these two time periods.**
**We have now changed the methods section ‘measuring the impact of hospital infection control’ to the following:**
***“Annual incidence rate ratios (IRR) were calculated using simulated data for one year pre- and post-intervention (T _0_ and T _1_ respectively) after first running the model to equilibrium. To aid comparison with reported infection data, we assumed the number of new infections (Y _in_) with and without a hospital link was proportional to the cumulative number of acquisitions (I _in_) in the hospital and community, respectively, in each of the two time periods:***
Equation 5
***Confidence intervals were calculated using 1000 Monte Carlo replicates on the assumption that the actual number of observed infections of each strain (Y _in_) followed a negative binomial distribution where Var(Y _in_) = µ+ µ ^2^/k, with k (the dispersion parameter) = µ/(*** ***- 1), with*** ***= 5, and assuming 1 in 10 carriage episodes acquired in hospital resulted in a reported infection. This was 1 in 50 for community-acquired episodes. Hence we allowed for differences in reporting rates in both settings as well as heterogeneity in case-mix affecting the likelihood of developing an infection. Then the IRR _in_ corresponded to the ratio of the number of new observed infections of strain i in population n in the year pre-intervention to the number in the first year post-intervention:”***
Equation 6
8. Interpretation of results about the dynamic after hospital infection control. To make the interpretation of Figure 3 more convincing, it would be important to disentangle what processes come from the community- to-community transmission, the hospital- to-community (ie community importations) transmission, the hospital- to-hospital transmission and the community-to-hospital (ie hospital importations) transmission. Could the authors present, in addition to Figure3, the incident cases coming from these different processes?
**We fully agree that it is helpful to show where the transmission events are actually occurring and this is what we are depicting in the top row of figure 3 - i.e. community-to-community transmission (dashed line) and hospital-to-hospital transmission (dotted line). The model assumes that community-to-hospital and hospital-to-community transmission events do not occur (we do not depict importation events of colonized patients to the hospital as these simply scale with community prevalence and would provide no new information). We apologies that the caption to figure 3 did not make this clear and we have now revised it**:
*“Trends in the incidence of new acquisitions (symptomatic and asymptomatic) and carriage prevalence for resistant and sensitive bacterial strains following a 10% stepwise improvement in hand hygiene compliance after one year from a baseline of 40%.* ***Incidence trends are depicted as transmission events following from community-to-community transmission (dashed line) and hospital-to-hospital transmission (dotted line).*** *As prevalence in the hospital represents only a small fraction of the overall prevalence (in hospital and community populations combined), the latter is almost identical to the community prevalence for both the resistant and sensitive bacterial strains.”*
9. The modelled hospital population is 1000 patients and 100 HCWs. The proposed model is deterministic. How would stochasticity impact the results?
**See the response to comment 1 reviewer #1**

 

Minor comments

1. Does R0 define the number of secondary cases of infection or colonisation? As transmission occurs through colonization my choice would go for that one but in the main text and Table 2 legend, the authors mention “infection”. In addition, as R0 is actually defined in a setting with already 40% hand hygiene at baseline, this is actually not the strict basic reproductive number of the bacteria. I therefore suggest naming it reproductive number (R) which seems more correct to me.
**In our model and the manuscript through out, R0 is considered the expected number of secondary cases in the hospital and community resulting from one colonised individual in a fully uncolonized and susceptible population at baseline hand hygiene rates of 40%, accounting for the possibility of readmissions while still colonized. The text in Table 2 has now been altered to reflect colonisation instead of infection. Moreover, we have altered R0 has now been changed to R _n_ (the net reproduction number) across the manuscript.**
2. Table1. hCWR and hCWS notations do not match with notations in the model depicted in fig1. Could the authors check they use the same notations?
**Many thanks for this sharp observation. We have now updated figure 1, which now matches our notation in Table 1.**

References

1. Ball J, Pike G, Griffiths P, Rafferty A, Murrells T. RN4CAST Nurse Survey in England. London; 2012.

2. Aiken L, Sloane D, Bruyneel L, Van Den Heede K, Griffi P, Busse R, et al. Nurse staffing and education and hospital mortality in nine European countries: a retrospective observational study. Lancet. 2014;383:1824–30.

3.Dingle K, Didelot X, Eyre D, Stoesser N, Golubchik T, Harding R, et al. Effects of control interventions on Clostridium difficile infection in England: an observational study. Lancet Infect Dis. 2017;

4.Albrich W, Harbarth S. Health-care workers: source, vector, or victim of MRSA ? Lancet Infect Dis. 2008;8(May):289–301.

5.Tong SYC, Holden MTG, Nickerson EK, Cooper BS, Cori A, Jombart T, et al. Genome sequencing defines phylogeny and spread of methicillin-resistant Staphylococcus aureus in a high transmission setting. 2015;1–9.

6. Wolinsky E, Lipsitz P, Mortimer E, Rammelkamp C. Acquisition of Staphylococci by newborns: direct versus indirect transmission. Lancet. 1960;276(7151):620–2.

7. Mortimer E, Wolinsky E, Gonzage A, Rammelkamp C. Role of Airborne Transmission in Staphyloccocal Infections. Br Med J. 1966;1:319–22.

8. Scanvic A, Denic L, Gaillon S, Giry P, Andremont A, Lucet J-C. Duration of Colonization by Methicillin-Resistant Staphylococcus aureus after Hospital Discharge and Risk Factors for Prolonged Carriage. Clin Infect Dis. 2001;32(May):1393–8.

9. van Kleef E, Kuijper E, Marc M, Cooper B. Clostridium difficile in England: can we stop washing our hands? Lancet Infect Dis. Elsevier Ltd; 2017;17(5):478–9.

10. Spicknall I, Foxman B, Marrs C, Eisenberg J. A modeling framework for the evolution and spread of antibiotic resistance: literature review and model categorization. Am J Epidemiol. 2013;178(4):508–20.

11. Cooper B, Medley G, Scott G. Preliminary analysis of the transmission dynamics of nosocomial infections: stochastic and management effects. J Hosp Infect. 1999;43(2):131–47.

12. Otto S, Day T. A Biologist’s Guide to Mathematical Modelling in Ecology and Evoluation. 1st ed. Oxfordshire: Princeton University Press; 2007.
